# Eco-evolutionary dynamics of temperate phages in periodic environments

**DOI:** 10.1093/ve/veaf019

**Published:** 2025-04-29

**Authors:** Tapan Goel, Stephen J Beckett, Joshua S Weitz

**Affiliations:** Department of Biology, University of Maryland, College Park, MD 20742, USA; Previous address: School of Biological Sciences, Georgia Institute of Technology, Atlanta, GA 30332, USA; Department of Biology, University of Maryland, College Park, MD 20742, USA; University of Maryland Institute for Health Computing, North Bethesda, MD 20852, USA; Previous address: School of Biological Sciences, Georgia Institute of Technology, Atlanta, GA 30332, USA; Department of Biology, University of Maryland, College Park, MD 20742, USA; University of Maryland Institute for Health Computing, North Bethesda, MD 20852, USA; Department of Physics, University of Maryland, College Park, MD 20742, USA; Previous address: School of Biological Sciences, Georgia Institute of Technology, Atlanta, GA 30332, USA; Institut de Biologie, École Normale Supérieure, 75005 Paris, France

## Abstract

Bacteriophages (viruses that exclusively infect bacteria) exhibit a continuum of infection mechanisms, including lysis and lysogeny in interactions with bacterial hosts. Recent work has demonstrated the short-term advantages of lysogeny over lysis in conditions of low host availability. Hence, temperate phage which can switch between lytic and lysogenic strategies—both stochastically and responsively—are hypothesized to have an evolutionary advantage in a broad range of conditions. However, the long-term advantages of lysogeny are not well understood, particularly when environmental conditions vary over time. To examine generalized drivers of viral strategies over the short- and long-term, we explore the eco-evolutionary dynamics of temperate viruses in periodic environments with varying levels of host availability and viral mortality. We use a nonlinear system of ordinary differential equations to simulate periodically-forced dynamics that separate a ‘within-growth’ phase and a ‘between-growth’ phase, in which a (potentially unequal) fraction of virus particles and lysogens survive. Using this ecological model and invasion analysis, we show and quantify how conflicts can arise between strategies in the short term that may favour lysis and strategies in the long term that may favour lysogeny. In doing so, we identify a wide range of conditions in which temperate strategies can outperform obligately lytic or lysogenic strategies. Finally, we demonstrate that temperate strategies can mitigate against the potential local extinction of viruses in stochastically fluctuating environments, providing further evidence of the eco-evolutionary benefits of being temperate.

## 1. Introduction

Temperate phages can have two alternative life cycles: lytic and lysogenic. The lytic life cycle involves phage entry into the host, followed by takeover of the host machinery to produce new phage and the eventual lysis of the host cell that releases new virions into the environment. These newly released virions can then go on to infect new hosts via horizontal transmission. The lysogenic life cycle involves the integration of the phage genome into the host genome as a prophage, leading to the formation of a lysogen that contains both the phage and the host genome (Lwoff, 1953; Golding, 2016; Zhang et al., 2021; Trinh et al., 2017). The phage genome reproduces when the lysogenized host cell divides, via vertical transmission. Lysogens can also induce, due to noisy gene regulation and/or cues (extracellular ones such as arbitrium (Aframian et al., 2022; Guler et al., 2024; Bernard et al., 2021) or intracellular ones such as stress signals (Ptashne, 1986)), and re-enter the lytic cycle.

Lysogeny provides direct and indirect benefits to both the phage and the host, in contrast to lysis, where the reproductive success of the phage comes at the expense of host mortality. In lysogeny, the reproductive success of the phage requires the reproductive success of the host, as the phage genome replicates with the host genome (Correa et al., 2021). More directly, lysogeny can confer superinfection immunity to the infected bacterium (Abedon, 2022; Howard-Varona et al., 2017; Bondy-Denomy et al., 2016), contribute to horizontal gene transfer and provide novel genetic material to the host (Harrison and Brockhurst, 2017) and, in cases where the bacterium itself is a pathogen to another organism, increase the pathogenicity of the bacterium (Bondy-Denomy and Davidson, 2014). Lysogens also contribute to phage diversity: integrated prophages can form coalitions with their hosts, allowing the coexistence of multiple types of phage with a single bacterial host species. This was demonstrated by (Basso et al., 2020), where lysogens of two genetically similar phages, immune to secondary infection from self but susceptible to lytic infections from heterospecific individuals, can both invade each other leading to coexistence.

There has been longstanding interest in the evolution and persistence of lysogeny as a strategy for viral reproduction (Stewart and Levin, 1984). At first glance, it would seem that lysogeny would have lower reproductive success than lysis: viral replication through lysogeny happens at the rate of cell division and only produces one new viral copy per cell division, while lysis is typically faster and produces several viral copies per lysis event. And yet, temperate phages are abundant in a variety of ecological settings (Howard-Varona et al., 2017): terrestrial (Williamson et al., 2007), host-associated (such as the mammalian gut) (Kim et al., 2011; Kim and Bae, 2018) and aquatic (Suttle, 2005; Wommack and Colwell, 2000). In the marine context, lysogen prevalence varies significantly whether estimated based on sequence analysis or prophage induction of isolates from a range of depths, spatial environments, and seasons (Tuttle and Buchan, 2020; Paul, 2008). Metagenomic analysis of dsDNA virus samples isolated from the North Pacific Subtropical Gyre reveals an increase in the prevalence of lysogeny with depth, which correlates with the decrease in host abundance (Luo et al., 2020). In polar environments, viral strategies shift from lytic infections during summer periods (when host and resources are abundant) to lysogenic infections during winter periods (when host and resources are relatively depleted) (Brum et al., 2016).

Theoretical models have been proposed to explain when and why lysogens persist and how their abundance varies with host and nutrient availability. The feast or famine hypothesis suggests that temperate strategies do better when host availability is low and extracellular viral mortality is high (Stewart and Levin, 1984). Formalizing the short-term benefits of viral strategies in terms of the basic reproduction number (i.e. the number of newly infected cells produced from a single infected cell in an otherwise susceptible population) has helped reveal how vertical transmission through lysogen replication outperforms lysis when host abundance is low and when extracellular phage mortality is high (Li et al., 2020; Wahl et al., 2019; Weitz et al., 2019). These models are consistent with observations of a negative correlation between lysogen abundance and host availability in marine environments (Brum et al., 2016; Luo et al., 2020). Alternative hypotheses, such as Piggyback-the-Winner, suggest the opposite: lysogeny rather than lysis increases with increasing host density (Knowles et al., 2016). However, counter to this assumption, current implementations of Piggyback-the-Winner models do not explicitly incorporate lysogeny and assume that productivity of viral lysis increases with host density (Weitz et al., 2017) and there is often conflicting (or lack of) evidence to support its claims (Weitz et al., 2017; Alrasheed et al., 2019). The roles and relative value of lytic vs. lysogenic strategies are still a matter of debate (see (Knowles et al., 2016; Weitz et al., 2017; Knowles and Rohwer, 2017; Coutinho et al., 2017; Alrasheed et al., 2019; Coutinho et al., 2019)) and may depend on specific ecological contexts. Fluctuations in the availability of hosts, the susceptibility of hosts, and resources available for cells and viruses to complete their life cycle suggest that temperate strategies may function as a means to ‘bet hedge’ against stochastic selection pressures (Maslov and Sneppen, 2015). Indeed, recent conceptual work has emphasized that infections may act along a continuum between lysis and latency (Correa et al., 2021).

**Figure 1.**
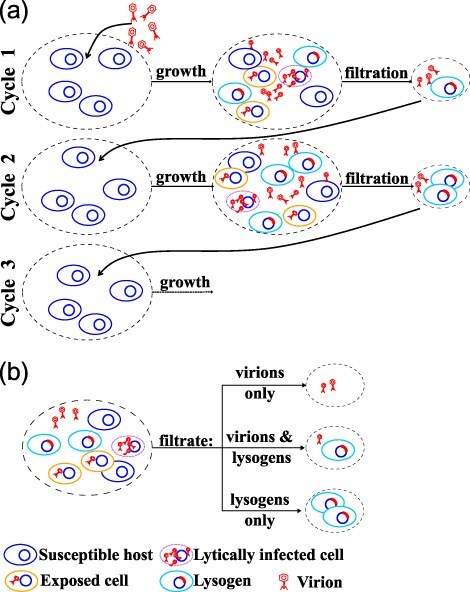
**Schematic of the serial passage model:** (a) In the first cycle, a fresh batch of nutrients and susceptible cells are inoculated with free virus particles. After a growth cycle, the system will have susceptible hosts, lysogens, lytically infected cells, and free virus particles. These cells (viruses) pass through a filter after which only a subset of susceptible cells, lysogens, and free viruses remain (in this case, lysogens and free viruses pass through the filter). This filtrate is now used to inoculate the new batch of host cells in cycle 2, and so on. The composition of the system at the end of each growth cycle changes, leading to changes in the composition of the filtrate, which in turn changes the initial conditions for the subsequent cycles. These cycles continue indefinitely or until the system reaches a steady state. (b) The filtration step can isolate virions alone (e.g. via size-dependent filtering), lysogens alone (e.g. via the use of phage-encoded antibiotic selection markers), or a mixture of the two.

Here, we study the long-term eco-evolutionary dynamics of temperate phages in environments with periodic fluctuations (Figure 1). Notably, periodic changes in host availability and viral decay, generated by external addition of nutrients and hosts, and differential dilution of virions and lysogens, can set conflicting short-term and long-term selection pressures on viral reproductive strategies. These conflicts of selection arise between strategies that do well in the short term (i.e. within a growth cycle) and those that do well in the long term (i.e. accounting for survival between growth cycles). Via a combination of pairwise invasion analysis and simulations of stochastically fluctuating environments, we identify a broad range of conditions where temperate strategies outcompete obligately lytic and obligately lysogenic strategies in the long term, providing further evidence of the eco-evolutionary benefits of being temperate.

## 2. Methods

### 2.1 Resource explicit SEILV model for the population dynamics of a one host-one virus system

We develop an eco-evolutionary model of phage-bacteria dynamics that consists of repeated serial passages. Each passage involves a growth cycle and a subsequent filtration step (see Figure 1). During the growth cycle, phage and bacteria interact in a well-mixed environment. The filtration step occurs at the end of the growth cycle. In the filtration step, different fractions of virions, lysogens, susceptible hosts, and resources that remain at the end of the growth cycle are introduced to a fresh batch of resources and susceptible hosts and the next growth cycle begins. This process of coupled growth cycles and filtration steps is then repeated over and over. This serial passage model represents a sequence of boom-and-bust periods, in which growth occurs during the boom period and viruses and/or cells degrade (potentially at different rates) during the bust periods.

We use a resource-explicit SEILV model (Li et al., 2020) to describe the phage-bacteria dynamics during a growth cycle. Susceptible hosts (*S*) consume resources (*R*) and replicate. Resource consumption and the consequent increase in host density are assumed to follow Monod kinetics (Monod, 1949) given by the function $\psi(R)$, where *µ*_max_ is the maximal growth rate of the cells and *R*_in_ is the resource density at which cells grow at half their maximal growth rate. Consumption of $1/e$ units of resources is required for cell division. Infectious virions (*V*) adsorb onto cells at adsorption rate *ϕ* and infect susceptible cells. Infected cells are initially classified as exposed cells (*E*). Exposed cells transition at rate *λ* and either form lysogens (*L*) with integration probability *p*, or form lytically infected cells (*I*) with probability $(1-p)$. Lysogens also consume resources and replicate, and they spontaneously induce a lytic fate at a per capita rate *γ*. The lytically infected cells lyse at a rate *η*, releasing *β* virus particles per lysis event. Susceptible, exposed, lytically infected, and lysogenic cells decay at per capita rates *d_S_*, *d_E_*, *d_I_*, and *d_L_*, respectively. Virions lose their infectivity at a per capita rate *m*. These ecological interactions are described by the following set of ordinary differential equations:


(1) $$ \begin{aligned} \dot{R} &= \overbrace{-e\psi(R) \left( S + E + I + L \right) }^{\text{nutrient uptake}} \\ \dot{S} &= \overbrace{\psi(R)S}^{\text{growth}} - \overbrace{\phi SV }^{\text{infection}} - \overbrace{d_S S}^{\text{decay}}\\ \dot{E} &= \overbrace{\phi SV}^{\text{infection}} - \overbrace{\lambda E}^{\text{transition}} - \overbrace{d_E E}^{\text{decay}}\\ \dot{I} &= \overbrace{(1-p) \lambda E}^{\text{lytic infection}} + \overbrace{\gamma L}^{\text{induction}} -\overbrace{\eta I}^{\text{lysis}} - \overbrace{d_I I}^{\text{decay}} \\ \dot{L} &= \overbrace{p \lambda E}^{\text{lysogenic infection}} + \overbrace{\psi(R)L}^{\text{growth}} - \overbrace{\gamma L}^{\text{induction}} -\overbrace{d_L L}^{\text{decay}} \\ \dot{V} &= \overbrace{\beta \eta I}^{\text{lysis}} - \overbrace{\phi V \left( S+E+I+L\right)}^{\text{adsorption}} - \overbrace{mV}^{\text{viral decay}} \\ \psi(R) &= \frac{\mu_{\text{max}} R}{R_{\text{in}}+R}. \end{aligned} $$

The definitions and values of the parameters in Equation 1 are provided in Table S1 and S2. For convenience, we use matrix notation to describe the state of the system and the filtration steps. The state of the system at time *t* after the beginning of *n*-th growth cycle is given by the six-dimensional vector $\vec{X}(n,t) = [R, \;S,\; E,\; I,\; L,\; V]^{\prime}$ where ($^{\prime}$) denotes a transpose. Each of the 6 state variables denote densities (here, we use units of $\text{mL}^{-1}$). The term $\vec{X}_i(n,t)$ refers to the *i*-th element of the vector $\vec{X}(n,t)$.

The filtration step is modelled via matrix multiplication:


(2) $$ \vec{F}_1(n) = \mathbf{Q_1} \vec{X}(n,t = T), $$

where $\vec{F}_1(n)$ is the filtrate at the end of the *n*-th growth cycle, *T* is the duration of the growth cycle and $\mathbf{Q_1} = \text{diag}(q_R, \; q_S, \; q_E,\;q_I,\;q_L,\;q_V)$ is the filtration matrix. $\mathbf{Q_1}$ is a diagonal matrix, with the *q_i_*’s being the fractions of each cell type (or virion or resource) that pass through the filter. For example, setting $q_V = 0.1$ means that 10 per cent of virions left at the end of the *n*-th cycle pass through the filter, setting $q_E = 0$ means that none of the exposed cells pass through the filter, and so on. We set $q_R = 0$ and $q_S = q_E = q_I = 0$, and vary *q_L_* and *q_V_* unless noted otherwise. The subsequent growth cycle then starts with the following initial condition:


(3) $$\begin{aligned} \vec{X}(n+1,t=0) &= \overbrace{[R_0,\;S_0,\;0,\;0,\;0,\;0]^{\prime}}^{\text{new host and resources}} + \overbrace{\vec{F}(n)}^{\text{filtrate}} \;\; \forall \;\; n \in \mathbb{N}, \end{aligned}$$

where $R_0 = 100\ \mu$g/mL and $S_0 = 10^7$ cells/mL are fixed densities of resources and susceptible hosts that are added at the beginning of each cycle. We use the following initial conditions for the first growth cycle:


(4) $$ \vec{X}(n =1,t = 0) = [R_0, \; S_0, \; 0,\; 0,\; 0, \; V_{0}]^{\prime}, $$

where $V_0 = 10^4$ virions/mL is an initial density of virions. In our work, the initial cell densities, *S*_0_, and viral densities, *V*_0_ are consistent with experimental studies (Marshall et al., 2022; Wang, 2006) and the value of the resource density, *R*_0_, corresponds to the glucose concentration of the Davis Mingioli (DM)100 culture medium in (Marshall et al., 2022).

The system of repeated cycles has reached a steady state when:


(5) $$ \vec{X}(n,t) = \vec{X}(n+1,t) \;\; \forall \;\; 0\leq t \leq T. $$

We denote the steady state growth cycle with $n^*$ and the state of the system at the beginning of the steady state growth cycle with $\vec{X}^*$:


(6) $$ \vec{X}^* = \vec{X}(n^*,t=0) = [R^*,\; S^*,\; E^*,\; I^*,\; L^*,\; V^*]^{\prime}. $$

$R^*$ , $S^*$, $E^*$, $I^*$, $L^*$, $V^*$ are the steady-state densities at the beginning of the cycle $n^*$.

### 2.2 Resource explicit SEILV model for the population dynamics of a one host-two virus system for evolutionary invasion analysis

We extend the model for the one host-one virus system to incorporate a second virus. We assume that the two virus types (denoted by *a* and *b*) differ only in the integration probabilities, i.e. probabilities of forming lysogens (*p*’s), and in the spontaneous induction rates (*γ*’s) of their respective lysogens. Each viral type is characterized by a $(p, \gamma)$ pair. We assume the presence of super-infection immunity—virions can adsorb onto infected cells, but they do not take over previously infected cells. The growth cycle dynamics are described by the following ordinary differential equations:


(7) $$ \begin{aligned} \dot{R} &= \overbrace{-e\psi(R) \left( S + E_a + E_b + I_a + I_b + L_a + L_b \right) }^{\text{nutrient uptake}} \\ \dot{S} &= \overbrace{\psi(R)S}^{\text{growth}} - \overbrace{\phi S(V_a + V_b)}^{\text{infection}} - \overbrace{d_S S}^{\text{decay}}\\ \dot{E}_a &= \overbrace{\phi SV_a}^{\text{infection}} - \overbrace{\lambda E_a}^{\text{transition}} - \overbrace{d_E E_a}^{\text{decay}}\\ \dot{E}_b &= \phi SV_b \quad - \lambda E_b - d_E E_b \\ \dot{I}_a &= \overbrace{(1-p_a) \lambda E_a}^{\text{lytic infection}} + \overbrace{\gamma_a L_a}^{\text{induction}} -\overbrace{\eta I_a}^{\text{lysis}} - \overbrace{d_I I_a}^{\text{decay}} \\ \dot{I}_b &= (1-p_b) \lambda E_b + \ \gamma_b L_b - \eta I_b - d_I I_b \\ \dot{L}_a &= \overbrace{p_a \lambda E_a}^{\text{lysogenic infection}} + \overbrace{\psi(R)L_a}^{\text{growth}} - \overbrace{\gamma_a L_a}^{\text{induction}} -\overbrace{d_L L_a}^{\text{decay}} \\ \dot{L}_b &= \quad \;\; p_b \lambda E_b \qquad + \psi(R)L_b - \; \gamma_b L_b \;\; - d_L L_b \\ \dot{V_a} &= \overbrace{\beta \eta I_a}^{\text{lysis}} - \overbrace{\phi V_a \left( S+E_a+E_b+I_a+I_b+L_a+L_b\right)}^{\text{adsorption}} - \overbrace{mV_a}^{\text{viral decay}} \\ \dot{V_b} &= \beta \eta I_b - \phi V_b \left( S+E_a+E_b+I_a+I_b+L_a+L_b\right) - \quad mV_b. \end{aligned} $$

The state of the system at time *t* after the start of the *n*-th growth cycle is given by a 10-dimensional vector $\vec{Y}(n,t) = [R,\;S,\;E_a,\;I_a,\;L_a,\;V_a,\;E_b,\;I_b,\;L_b,\;V_b]^{\prime}$. $\vec{Y}_i(n,t)$ denotes the *i*-th element of the vector $\vec{Y}(n,t)$. Similar to the model for the one host-one virus system, the filtration step at the end of the *n*-th and initial condition for the $(n+1)$-th cycle are defined by:


$$ \begin{aligned} \vec{F}_2(n) &= \mathbf{Q_2}\vec{Y}(n,t = T),\\ \vec{Y}(n+1,t = 0) &= [R_0,\;S_0,\;0,\;0,\;0,\;0,\;0,\;0,\;0,\;0]^{\prime}+ \vec{F}_2(n), \\ \end{aligned} $$

where $\mathbf{Q_2} = \text{diag}(q_R, \; q_S, \;q_E,\;q_I,\;q_L,\;q_V,\;q_E,\;q_I,\;q_L,\;q_V)$ is a filtration matrix similar to $\mathbf{Q_1}$.

We use this model to analyse whether a mutant (represented by virus type *b*), appearing at a low initial frequency, with traits $(p_b,\gamma_b)$ can invade a population where the resident (represented by virus type *a*) with traits $(p_a, \gamma_a)$ is at steady state with the host. To simulate this scenario, we first let the one host-one virus system with virus type *a* reach steady state $\vec{X}^*_a = [R^*,\; S^*,\; E_a^*,\; I_a^*,\; L_a^*,\; V_a^*]^{\prime}$. We then initialize the one host-two virus system with the initial condition:


(9) $$\begin{aligned} \vec{Y}(n=1,t = 0) &= [R_0, \;S_0, \;E_a^*,\;I_a^*,\;L_a^*,\;V_a^*,\;M\frac{q_E}{q},\; M\frac{q_I}{q},\;M\frac{q_L}{q},\;M\frac{q_V}{q}]^{\prime}. \end{aligned}$$

*M* is the (small) initial total density of viral genome copies of the mutant and $q = q_E+q_I+q_L+q_V$ is a normalization factor. The normalization ensures that irrespective of the filtration matrix, the total density of mutant viral genome copies introduced into the system is always *M*. Out of the *M* mutant viral genome copies, $\frac{q_L}{q}$ of them are in the form of lysogens, $\frac{q_V}{q}$ of them are in the form of virions, and so on. We say that virus type *b* invades successfully if, in the first few cycles, the total genome density of virus type *b* at the beginning of successive growth cycles increases above *M*. On the other hand, if the total genome density of virus type *b* at the beginning of successive growth cycles decreases below *M*, we say that the invasion failed (see section 2.3 for details of the invasion criterion).

### 2.3 Simulation details

Mathematically, Equation 5 defines the condition for the steady state of the one host-one virus system. Numerically, however, we define the system to have reached a steady state $\vec{X}^*$ by the $n^*$-th growth cycle if, in ten consecutive growth cycles starting at $n^*$, the initial densities of cells (and virions) in successive growth cycle are within a threshold *ϵ* of each other. In practice, we require:


$$\begin{aligned} \| \vec{X}_i(n+1,t = 0) &- \vec{X}_i(n,t = 0) \| \leq \epsilon \;\; \;\; \forall \;\; i \in \{1,...,6\} \;\;\text{and} \;\; \forall \;\; n^* \leq n \leq n^*+10. \end{aligned}$$

$$\begin{aligned} \vec{X}^* &= \vec{X}(n^*,t = 0). \end{aligned}$$

Here, $\vec{X}_i$ is the *i*-th element of the vector $\vec{X}$ (i.e. $\vec{X}_2$ is the density of susceptible cells, $\vec{X}_6$ is the density of virions, and so on) and *ϵ* is the critical density threshold. In this study, we assume that the total volume of the system is 1000 mL. Therefore, densities below *ϵ* = 0.001/mL correspond to less than one cell (or virion) in the entire system. So any differences in densities below *ϵ* are not meaningful in practice.

In the invasion analysis we use $M = 10\epsilon$ which corresponds to 10 viral genome copies in the entire system, such that the mutant appears at a much lower frequency compared to the resident virus. Further, we only perform invasion analysis when there are more than 100 copies of the resident viral genome, i.e. $E_a^* + I_a^* + L_a^* + V_a^* \geq 100 \epsilon$ (where $E_a^*$, $I_a^*$, $L_a^*$ and $V_a^*$ are defined in Equation 9). This is to ensure that the resident is present at a much higher frequency than the mutant and, to ensure that the invasion dynamics are not driven entirely by transients.

To assess if virus type *b* invades successfully, we evaluate whether the total viral genome density of virus type *b* has increased or decreased after *k* cycles. We choose *k* = 10 in practice to reduce the impact of transients. If, after *k* cycles, the total viral genome density of virus type *b* is greater than its initial density *M* and is continuing to increase, then the invasion is classified as successful. On the other hand, if the total viral genome density of virus type *b* is less than *M* and continues to decrease after *k* cycles, the invasion is classified as unsuccessful. If neither set of conditions is met, we repeat this process of simulating *k* cycles until either the condition for invasion success or the condition for invasion failure is met.

To investigate the dynamics of a one-host one-virus system under stochastic filtration conditions for lysogens and virions, we modify our definition of $\mathbf{Q_1}$ to represent a stochastic filtration matrix where *q_L_* and *q_V_* are drawn independently from log-uniform distributions over spans $[10^{k_L},10^{-1}]$ and $[10^{k_V},10^{-1}]$, respectively, at the end of each growth cycle *n*, such that $\mathbf{Q_1}(n) = \text{diag}(q_R = 0, \; q_S = 0, \; q_E = 0,\;q_I = 0,\;q_L,\;q_V)$ . To evaluate how this stochasticity impacts the long-term success of viral strategies we simulate 100 consecutive 24-hr growth cycles as described by Equation 1, Equation 2 and Equation 3. For each of the viral strategies investigated, we start with the initial condition $\vec{X}(n = 1, t = 0) = [R_0, \; S_0,\; 0,\;0,\;0,\;V_0]$ and track the population over 100 growth cycles. If, at the beginning of any cycle, both the density of virions and the density of lysogens fall below the critical density threshold *ϵ*, we assume that the virus is eliminated. For each viral strategy and each pair of values of (*k_L_*,*k_V_*), we create an ensemble of 100 stochastic simulations. We then define the probability of survival as the fraction of realizations in which the virus does not perish by the end of 100 cycles.

Simulation parameters, unless stated otherwise, are provided in Table S1 and Table S2. All parameters, except for the cell death rates and viral decay rates in Table S1 were taken from (Shivam et al., 2022). The cell death rates and viral decay rates in Table S1 are about half of the rates provided in (Shivam et al., 2022). All code was written in MATLAB R2022b and MATLAB R2023b, and is available for download at https://github.com/tapangoel1994/EcoEvoDynamicsInPeriodicEnvironments and archived on Zenodo at (Goel et al., 2025).

## 3. Results

### 3.1 Virus-host dynamics in the short-term

**Figure 2.**
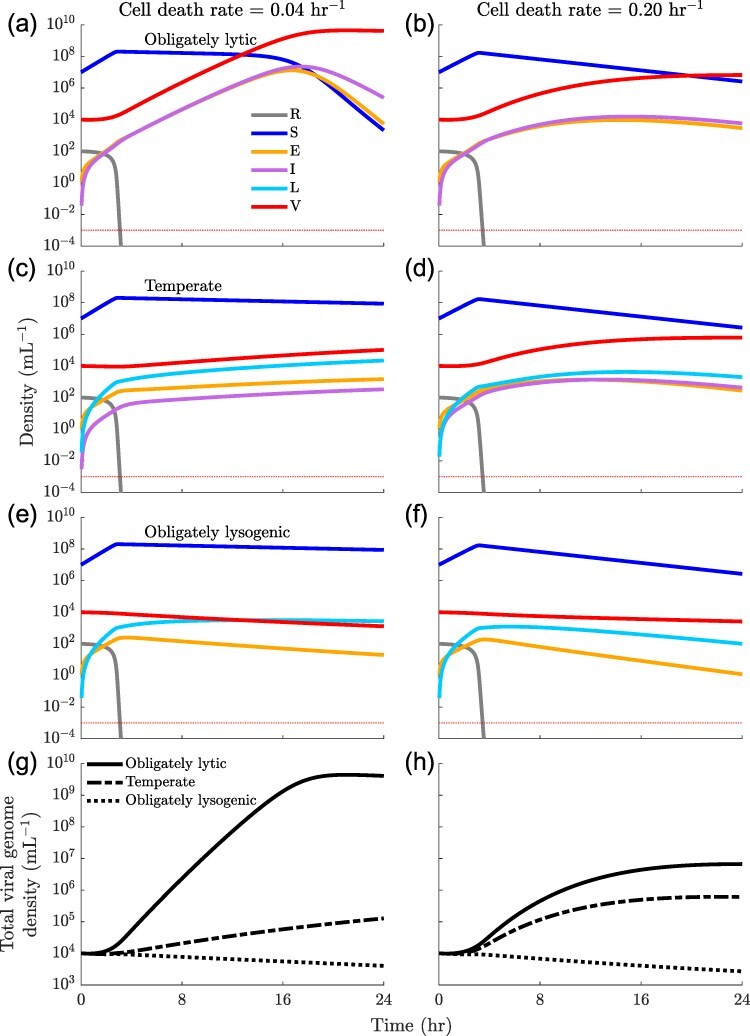
**Short-term population dynamics:** Population dynamics during a 24-hr growth cycle for (a, b) obligately lytic $(p = 0,\gamma=0\ \text{hr}^{-1})$, (c, d) temperate ($p = 0.92,\gamma=0.006\ \text{hr}^{-1}$ in (c); $p = 0.50,\gamma=0.083\ \text{hr}^{-1}$ in (d)) and (e, f) lysogenic $(p = 1,\gamma=0\ \text{hr}^{-1})$ viruses. (g, h) Total viral genome density $(E+I+L+V)$ for the obligately lytic (solid line), temperate (dashed line), and obligately lysogenic (dotted line) viruses. All other simulation parameters are given in Table S1 and Table S2. The cell death rates ($d_S, d_E, d_I, d_L$) are $0.04\ \text{hr}^{-1}$ in a, c, e, and g and, $0.2\ \text{hr}^{-1}$ in b, d, f, and h. Dotted horizontal red line in a-f represents the critical density threshold $\epsilon = 10^{-3}$ mL^−1^.

We simulate the SEILV model with one virus and one bacterial host. Figure 2 shows the population dynamics for the first 24-hr growth cycle for three different virus types, each characterized by an induction rate *γ* and integration probability *p*: (i) obligately lytic ($p = 0, \gamma = 0\ \text{hr}^{-1}$), (ii) temperate ($p = 0.92,\gamma = 0.006\ \text{hr}^{-1}$) and ($p = 0.50,\gamma = 0.083\ \text{hr}^{-1}$), and (iii) obligately lysogenic ($p = 1,\gamma = 0\ \text{hr}^{-1}$). The obligately lysogenic virus represents an extreme case where the virus never reenters its free virus particle life stage and therefore represents an evolutionary dead end where the viral genome is potentially domesticated by the host. We include this obligately lysogenic strategy in order to evaluate a range of life history strategies—including those between extremal strategies. We also consider two different scenarios to assess the robustness of our findings: when the cell death rate is the same as the viral decay rate, and when the cell death rate is much higher than the viral decay rate. Across the three viral strategies and cell death rates considered here, in the first four hours the host density increases since cell division outpaces infection. As the density of virions increases and the density of resources decreases, the susceptible population begins to decline due to the increased rate of infection and the slowing down of cell division (Figure 2). The obligately lytic virus exploits the high density of susceptible hosts and produces new virions through lytic infections, which go on to infect other susceptible hosts. As a result, the virion density increases, slowly at first due to the lack of virions, then quickly due to an abundance of both hosts and virions, and slowly again due to the lack of susceptible hosts (Figure 2a, b). This is true in the low cell death rate case, as well as the high cell death rate case. The obligately lysogenic virus, on the other hand, only produces lysogens upon infection, which in turn only reproduce by cell division. First, the lysogen population grows quickly due to lysogenic infections and cell division and then begins to decline due to cell death (Figure 2e, f). The temperate virus produces both virions and lysogens, albeit at slower rates than the obligately lytic and obligately lysogenic viruses, respectively (Figure 2c, d). Temperate lysogens produce new lysogens through cell division, and they also produce new virions through induction. These new virions, in turn, can go on to infect additional cells, some of which may become lysogens. As a result, by the end of the growth cycle, the temperate virus can produce more lysogens than the obligately lysogenic virus. So, for a given environmental context, different viral strategies lead to different ecological population dynamics and consequently, different reproductive fitness as measured in terms of the total number of viral genomes produced during a single growth cycle. As shown in Figure 2g, h, the obligately lytic virus produces the largest number of viral genome copies (the sum of viral genomes inside infected cells and free virions) over the 24-hr growth phase, followed by the temperate and lastly obligately lysogenic viruses. Therefore, when only considering variation in integration and induction over the timescale of a single growth cycle (24 hr) and given abundant available host cells, the obligately lytic virus has greater reproductive fitness than the temperate virus, which in turn has greater reproductive fitness than the obligately lysogenic virus.

### 3.2 Virus-host dynamics in the long-term

We investigate cycle-to-cycle dynamics for different viral strategies in response to different filtration conditions. We consider filtration conditions where (i) only virions, (ii) a mixture of virions and lysogens, and (iii) only lysogens pass from one cycle to the next. The different filtration conditions favour different viral strategies over the long term: when only virions pass from cycle to cycle, we expect strategies that favour lysis to persist in the long term—as lysogens are removed at the end of every cycle. On the other hand, when only lysogens pass through the filter, we expect strategies that include lysogeny to persist—as free viruses do not pass to the next growth cycle. However, it is unclear how cycle-to-cycle filtration interacts with nonlinear ecological dynamics during the growth cycle to determine the success of viral reproductive strategies.

**Figure 3.**
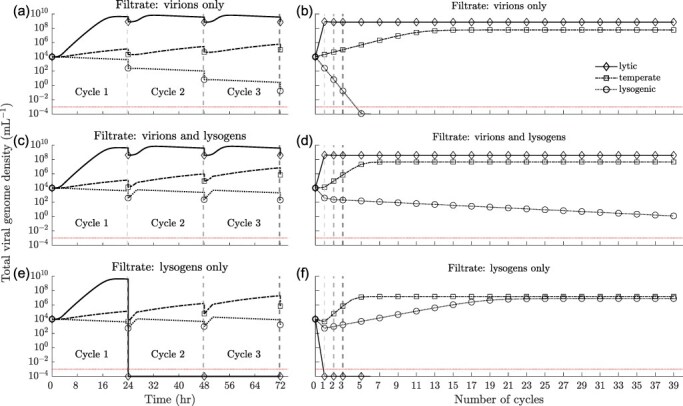
**Long-term population dynamics:** Total viral genome densities over the first three 24-hr growth cycles for obligately lytic $(p = 0, \gamma=0\ \text{hr}^{-1})$ ($\diamond$), temperate $(p = 0.92,\gamma=0.006\ \text{hr}^{-1})$ ($\square$) and lysogenic $(p = 1,\gamma=0\ \text{hr}^{-1})$ ($\circ$) viruses when (a) only virions ($q_V = 0.2$), (c) lysogens and virions ($q_V = 0.1, q_L = 0.1$), and (e) only lysogens ($q_L = 0.2$) pass through the filter. Total viral genome densities at the beginning of each growth cycle for obligately lytic $(p = 0, \gamma=0\ \text{hr}^{-1})$ ($\diamond$), temperate $(p = 0.92, \gamma=0.006\ \text{hr}^{-1})$ ($\square$) and lysogenic $(p = 1,\gamma=0\ \text{hr}^{-1})$ ($\circ$) viruses when (b) only virions, (d) lysogens and virions, and (f) only lysogens pass through the filter. The dashed vertical lines at the 24, 48, and 72 hr marks in plots a, c, and e correspond to the dashed vertical lines at cycle numbers 1, 2, and 3 in plots b, d, and f, respectively. All other simulation parameters are given in Table S1 and Table S2. Dotted horizontal red line in a–f represents the critical density threshold $\epsilon = 10^{-3}$ mL^−1^.

First, we consider what happens when only virions pass from one cycle to the next. This scenario simulates the serial passage protocol commonly used in experimental evolution studies of phage virulence. Figure 3a shows the dynamics of total viral genome copies through the first three growth cycles, and Figure 3b shows the longer-term cycle-to-cycle dynamics. In the first growth cycle, the obligately lytic virus produces the largest number of viral genome copies, all in the form of virions (Figure S1a). As a result, the obligately lytic virus transfers the maximum number of viral genome copies to the next cycle. However, temperate and lysogenic viruses produce fewer total viral genome copies (Figure S1c, e). Further, only some of these new genome copies are virions (the rest are lysogens) (Figure S1c, e). As a result, temperate and obligately lysogenic viruses transfer fewer viral genome copies to the next cycle (Figure 3a).

As this process repeats from cycle to cycle, we observe that the initial density of viral genome copies across growth cycles stops changing over time (Figure 3b). In fact, after 20 growth cycles, the cycle-to-cycle population dynamics for each viral strategy converge (up to the critical density threshold *ϵ*) (Figure S1), consistent with the system having reached steady state (see section 2.3 for details). At steady state, the obligately lytic virus passes on the highest number of viral genome copies from one cycle to the next (Figure 3b). The obligately lysogenic virus, on the other hand, does not pass any viral genomes to the next cycle: the obligately lysogenic virus does not produce free virions (Figure S1e, f) and therefore the total viral genome density decreases from cycle to cycle (Figure 3a) until there are no viral genomes left (Figure 3b). The temperate virus, however, persists in the population since virions are produced by both, lytic infection and lysogen induction. So, when virions alone are filtered through across the growth cycle, the obligately lytic virus and the temperate virus persist over the long term, while the density of the obligately lysogenic virus rapidly diminishes until it is eliminated after five growth cycles.

Next, we consider what happens when only lysogens pass from one cycle to the next (this corresponds to cases where extracellular virions might rapidly decay or degrade outside of growth periods) (Figure 3e, f). As in the previous scenario, we find that the system reaches a steady state after a few growth cycles (Figure 3f, Figure S2). However, in this scenario the obligately lytic virus does not persist in the long term. Even though the obligately lytic virus produces the most viral genomes in the first growth cycle, none of these genome copies make it to the second cycle since only lysogens pass through the filter (Figure 3e, Figure S2a, b). As a result, the obligately lytic virus perishes after the first filtration (Figure S2a). The obligately lysogenic and temperate viruses, on the other hand, generate lysogens which can pass on the viral genome from one growth cycle to the next and therefore continue to persist past the first growth cycle (Figure S2c-f). The obligately lysogenic virus persists due to the cell division of new lysogens in a single growth cycle exceeding losses due to lysogen decay and filtration. The temperate virus persists given the balance between lysis which produces more virions that infect host cells during the growth cycle with the direct generation of lysogens that pass the viral genome (as a prophage) across growth cycles (Figure S2c, d).

We also consider what happens when both lysogens and virions pass from cycle to cycle (Figure 3c, d). In this scenario, the obligately lytic virus persists because of the production and passaging of virions, while the temperate virus persists due to the production and passaging of virions and lysogens (Figure S3). The obligately lysogenic virus persists at a very low density (Figure S4) because it is unable to generate sufficient viral copies during the growth cycle to compensate for filtration at the end of the cycle.

Comparing the long-term population outcomes for the same viral strategy across different filtration conditions, we find that the total initial viral genome density at steady state for the obligately lytic virus is lower when both lysogens and virions pass through, compared to when only virions pass between growth cycles (Figure S4). Similarly, the total viral genome density in steady state for the temperate virus is highest when only virions pass through, followed by when virions and lysogens pass through, and lowest when only lysogens pass through (Figure S4). On the other hand, the total density of the viral genome in steady state for the obligately lysogenic virus is highest when only lysogens pass through the filter, is very small when lysogens and virions pass through the filter, and is zero when only virions pass through the filter (Figure S4).

When cell death rates are higher (Figure S5), we still find that the obligately lytic virus produces the largest number of viral genome copies in the first growth cycle. When virions alone or a combination of virions and lysogens are passed (Figure S5), the obligate lytic virus also produces the maximum number of viral genome copies in the long term (Figure S6, Figure S8). However, when lysogens alone are passaged, the obligately lytic virus perishes after the first cycle (Figure S7). On the other hand, the temperate virus persists across the different filtration scenarios (Figure S5-S8) due to the balance between virion and lysogen production. Contrary to what we observe in the low cell death rate cases discussed above, the obligately lysogenic virus always dies out when the cell death rate is high, irrespective of the filtration condition (Figure S5-S9). This happens because in the absence of resources during the later part of the growth cycles, lysogens produced by the obligately lysogenic virus are unable to reproduce. In the absence of cell replication, exponential cell death leads to the depletion of lysogens.

These results demonstrate that the long-term success of viral strategies depends on the interplay between viral reproductive strategy, ecological feedback during growth cycles (i.e. boom periods), and the relative rates of mortality for the different stages in the viral life cycle during filtration (i.e. bust periods).

### 3.3 Temperate strategies maximize steady-state viral genome density under conflicting short-term and long-term selection pressures

As we showed in the previous section, depending on the filtration conditions, the success of viral strategies may be discordant between the long term vs. the short term. Analyzing the fitness of different viral life history strategies requires comparing population dynamics over the long term. We do so by comparing the total viral genome densities at the beginning of steady-state growth cycles across the continuum of viral strategies in the $(p,\gamma)$ space, for different filtration conditions and cycle periods.

**Figure 4.**
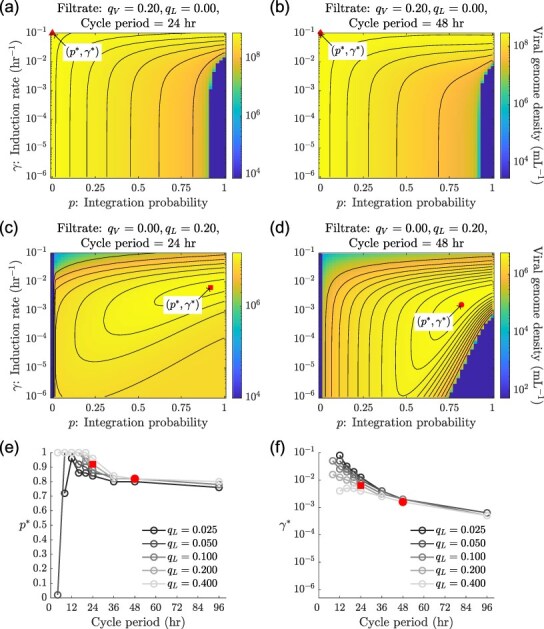
**Steady states for different long-term selection pressures:** Heatmaps of the density of viral genome copies at the beginning of a steady state growth cycle across different induction rates and integration probabilities when only virions ($q_V=0.2$) are passaged every (a) 24 hr and every (b) 48 hr, and when only lysogens ($q_L=0.2$) are passaged every (c) 24 hr and every (d) 48-hr. The set $(p^*,\gamma^*)$ denotes the strategies that maximize the steady-state viral genome density for the given filtration and cycle period conditions. (e) Integration probabilities and (f) induction rates of strategies that maximize the steady state total viral genome density as a function of cycle period. Induction rates are not plotted for strategies where $p^* = 0$, since induction is not meaningful when no lysogens can be created. Line colours correspond to the fraction of lysogens that pass from one cycle to the next. Fraction of virions in the filtrate (*q_V_*) is set to zero. The red squares in c, e, and f correspond to the same strategy for the same filtration and cycle period conditions. Similarly, the red circles in d, e, and f correspond to the same strategy. All other simulation parameters can be found in Table S1 and Table S2.

We first consider what happens when only virions are passed from one 24-hr growth cycle to the next. The obligately lytic strategies (*p* = 0) produce the maximum number of viral genome copies at steady state (Figure 4a). Viruses with more temperate strategies (i.e. *p* > 0) also persist at steady state but do not produce as many viral genome copies (Figure 4a). We find similar results when the cycle period is increased to 48 hr—when there are only virions in the filtrate, the obligate lytic strategy maximizes the total viral genome density at steady state (Figure 4b).

On the other hand, when only lysogens are passaged across the 24-hr cycles, obligately lytic viruses do not persist at steady state (Figure 4c). However, temperate and lysogenic viruses persist. Within the continuum of temperate and obligately lysogenic viral strategies, some strategies do better than others: we find that an intermediate strategy $(p^* = 0.92, \gamma^* = 0.0063\ \text{hr}^{-1})$ maximizes the steady-state viral genome density (Figure 4c). Similarly, for 48-hr growth cycles, a temperate strategy $(p^* = 0.82, \gamma^* = 0.0016\ \text{hr}^{-1})$ maximizes the total viral genome density at steady state (Figure 4d). We also find that viruses that always form lysogens, i.e. viruses with *p* = 1, must have a minimum induction rate to persist in the long-term, as demonstrated by the transition between the purple to the yellow region (Figure 4d).

The success of a temperate strategy in scenarios where lysogens alone are passaged can be explained qualitatively by looking at the conflict between the short- and long-term selection pressures set by the host input and by filtration. Since lysogens alone carry the viral genome from cycle to cycle, the strategy that maintains the highest lysogen density at the end of a growth cycle will maximize the viral genome density at steady state. There are primarily two ways in which new lysogens are generated: lysogens reproducing to make new lysogens ($L \rightarrow L$) and free virions (produced by lysis) infecting susceptible cells, which then form lysogens ($I \rightarrow V \rightarrow E \rightarrow L$) (see (Weitz et al., 2019) for related analysis of temperate life cycles). The first path is only available when resources are present in the system, while the second path is only available when there are susceptible cells in the system. The production of lysogens along the second path depends on having a large number of lytically infected cells, which requires large *γ* for the $L \rightarrow I$ transition and low *p* for the $E \rightarrow I$ transition. But, maximizing the number of lysogens produced also requires low *γ* to prevent the $L \rightarrow I$ transition and high *p* for the $E \rightarrow L$ transition. An intermediate temperate strategy that balances this trade-off generates the highest density of lysogens (and, therefore, viral genome copies) at steady state. We obtain similar qualitative results when cell death rates are high (Figure S10).

We plot the viral genome maximizing strategies $(p^*,\gamma^*)$ for different cycle periods and different amounts of lysogens alone passing through the filter and find that $p^*$ and $\gamma^*$ show the same increasing and decreasing trends with changing filtration conditions and cycle periods (Figure 4e, f). As $p^*$ increases or decreases with cycle period and/or the fraction of lysogens that pass from one cycle to the next, so does $\gamma^*$. The positive correlation between $p^*$ and $\gamma^*$ points to a trade-off between the formation and maintenance of lysogens. However, when only virions pass from cycle to cycle, for all sampled cycle periods and the amount of virions passaged, obligately lytic strategies always maximize the steady-state viral genome density. This result agrees with expectations that when both short-term and long-term selection pressures favour lysis, obligately lytic strategies succeed but when selection pressures act in opposing directions, temperate strategies maximize viral genome densities.

### 3.4 Temperate strategies are evolutionarily stable when short-term and long-term selection pressures act in opposite directions

We utilize invasion analysis and pairwise invasibility plots (PIP) (Brännström et al., 2013) to assess the relative long-term fitness of viral strategies. First, we allow a one-host, one-virus (the resident virus) system to reach steady state, then add a second virus type (the mutant) with a different life history strategy at very low density and evaluate whether the population of the second virus increases from cycle to cycle over the course of the first few growth cycles (see section 2.3 for details). To analyse invasions for a fixed cycle period and filtration condition, we fix the induction rate of the resident and the mutant to be $\gamma = \gamma ^*$ but allow them to have different integration probabilities.

**Figure 5.**
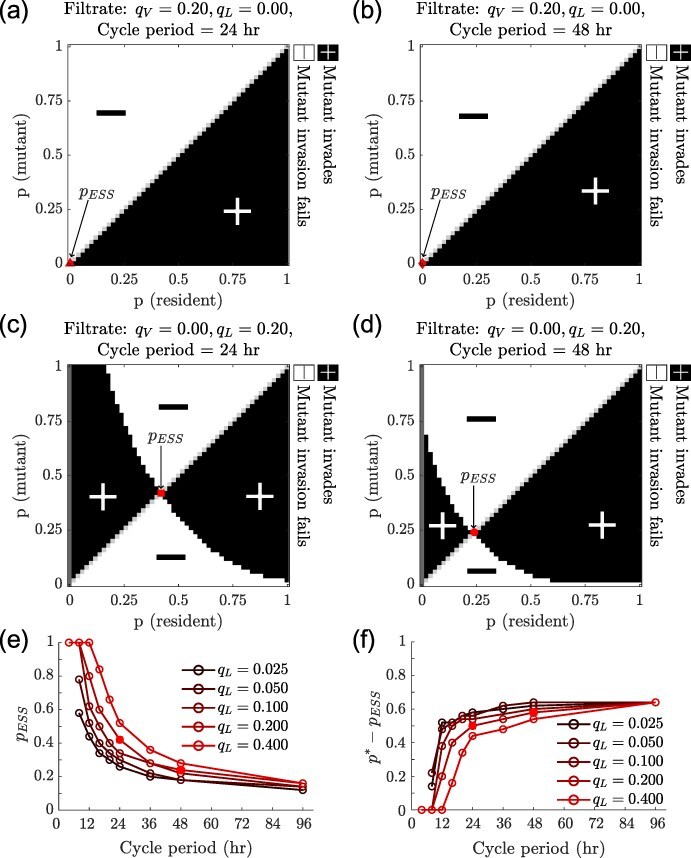
**Invasion analysis:** PIP when only virions ($q_V=0.2$) are passaged every (a) 24 hr and every (b) 48 hr, and when only lysogens ($q_L=0.2$) are passaged every (c) 24 hr and every (d) 48 hr. Red markers indicate the evolutionarily stable integration probability (*p*_ESS_) for fixed induction rates corresponding to those that maximize viral genome density at steady state ($\gamma = \gamma^*$) for each condition. Dark grey regions correspond to integration probabilities at which the resident virus does not persist in the one host-one virus system (see section 2.3 for details). Light grey marks regions where the resident and mutant have identical life history strategies. (e) Evolutionarily stable integration probability as a function of cycle period. Colours correspond to the fraction of lysogens that pass from one cycle to the next. The ESS for each condition is calculated by setting the induction rate to be the viral genome density maximizing the induction rate for that condition. (f) Difference between the steady-state viral genome maximizing integration probability and the evolutionarily stable integration probability for different cycle periods and filtration conditions. The red squares in c, e, and f correspond to the same strategy for the same filtration and cycle period conditions. Similarly, the red circles in d, e, and f correspond to the same strategy. All other simulation parameters can be found in Table S1 and Table S2.

Figure 5a is the PIP for the scenario in which only virions pass from cycle to cycle and the cycle period is 24 hr. Here, we see that virus types with more lytic traits (i.e. lower integration probabilities) always invade residents with more lysogenic traits (i.e. higher integration probabilities). Thus, we expect viral populations to evolve towards lower and lower integration probabilities, eventually reaching $p \rightarrow 0$, in agreement with the one-host one-virus analysis in Figure 4a. Lysis is favoured in the short term since a high density of susceptible hosts is added in each growth cycle, and lysis is also favoured in the long term since virions (produced by lysis) transfer the viral genome between growth cycles. We obtain the same result (when only virions are passaged) over 48-hr cycles instead of 24-hr cycles (Figure 5b).

On the other hand, when only lysogens pass from cycle to cycle over 24-hr cycles, we find that a temperate strategy ($p_{\text{ESS}}=0.42$) has the highest invasion fitness (Figure 5c). The region $p{(\text{resident})} = 0.0$ in Figure 5c is infeasible as it corresponds to an obligate lytic strategy, which cannot persist when only lysogens are filtered (see section 2.3 for details). In the feasible region $p({\text{resident}}) > 0.0$, we find an evolutionarily stable strategy (ESS) given by $p_{\text{ESS}} = 0.42$ (Figure 5c) that cannot be invaded by a viral mutant using any alternative integration probability. This *p*_ESS_ strategy is also convergence stable. Assuming the evolutionary timescale is longer than the ecological timescale, and that mutations are both rare and lead to small changes in the integration phenotype, one would expect a single temperate phage in the system with an integration probability $p = p_{\text{ESS}}$. We obtain qualitatively similar results for the high cell death rate case (Figure S11), demonstrating the robustness of the idea that conflicting selection pressures can lead to the selection of temperate phenotypes. Similar results have been demonstrated in the context of a host-limited model of temperate phages (Boldin, 2022).

Analyzing scenarios with different cycle periods and fractions of lysogens in the filtrate, we find that for a given filtration fraction, the *p*_ESS_ decreases with cycle period (Figure 5e). This link between growth cycle duration and *p*_ESS_ can be explained by changes in the relative contributions of the vertical ($L \rightarrow L$) and the horizontal ($ I \rightarrow V \rightarrow E \rightarrow L$) transmission modes in production lysogens over time. Initially, when resources are present in the system, the vertical mode of transmission produces lysogens. Once resources are depleted and cell division drops to effectively zero, new lysogens are only produced through infection of susceptible cells via horizontal transmission. Meanwhile, existing lysogens are depleted due to cell death and induction. Since the resources are depleted early on during the growth cycle, the potential for lysogen production via strictly vertical transmission is limited. As growth cycle duration increases, the production of lysogens relies on horizontal transmission for a larger fraction of the growth cycle. Therefore, as the growth cycle duration increases, strategies with a lower integration probability produce more offspring since they favour horizontal transmission.

The conflict between the short- and long-term selection pressures, observed when only lysogens pass through the filter, is also observed by comparing the evolutionary stable strategy (ESS) given by $(p_{\text{ESS}})$ and the strategy that maximizes the steady-state viral genome density $(p^*)$. We find, for the same induction rate $\gamma^*$, that $p_{\text{ESS}} < p^*$ across cycle periods and lysogen fractions (Figure 5f). The ESS has more lytic traits than the strategy that maximizes the steady-state viral genome density. This difference arises from selection operating at two different timescales. While the virus with integration probability $p^*$ would produce more lysogens in isolation than the virus with integration probability *p*_ESS_, when the two viruses compete, the virus with the evolutionarily stable viral strategy (*p*_ESS_) infects more hosts in the initial part of a growth cycle as it produces more lytic infections during each cycle. Consequently, there are fewer susceptible hosts available to the strategy with integration probability $p^*$. Therefore, the ESS does better in the short term and eventually outcompetes the strategy that maximizes the density of viral genomes passaged from cycle to cycle at steady state (Figure S12).

### 3.5 Being temperate provides protection against environmental stochasticity

**Figure 6.**
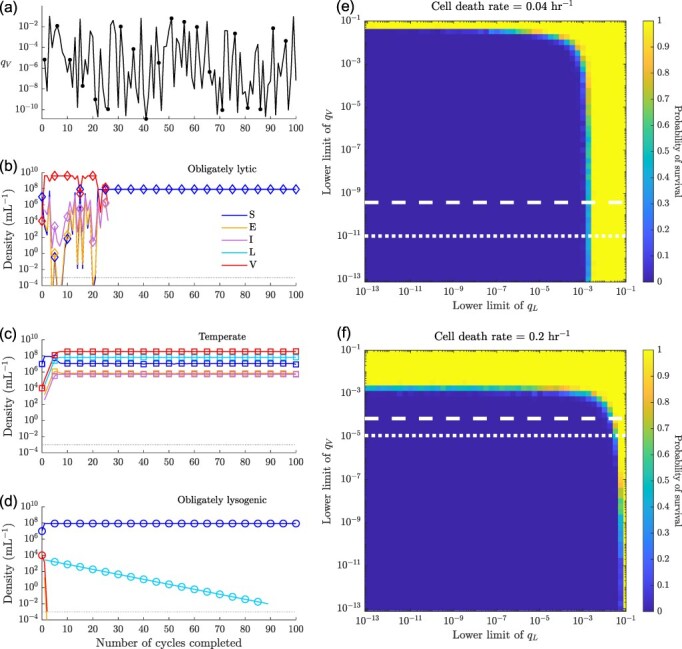
**Population dynamics with stochastic filtration:** (a) *q_V_* values for 100 growth cycles, chosen from a log uniform distribution over the span $[10^{-10},10^{-1}]$. Population densities at the end of each growth cycle for (b) obligately lytic ($p = 0,\gamma = 0\ \text{hr}^{-1}$), (c) temperate ($p = 0.92, \gamma = 0.006\ \text{hr}^{-1}$) and (d) obligately lysogenic ($p = 1, \gamma = 0\ \text{hr}^{-1}$) viruses across hundred 24-hr growth cycles. In total, 10 per cent lysogens ($q_L = 0.1$) are passaged from one cycle to the next in every case, but the virion fraction passaged from cycle to cycle is set by *q_V_* shown in (a). Markers at every 5^th^ cycle denote that the lines represent discrete cycle-to-cycle dynamics and not continuous-time dynamics. Heatmaps showing the probability that temperate viruses with strategies (e) $(p = 0.92, \gamma = 0.006 \ \text{hr}^{-1})$ and (f) $(p = 0.5, \gamma = 0.083\ \text{hr}^{-1})$ survive 100 growth cycles where the filtration parameters *q_L_* and *q_V_* are drawn independently from log-uniform distributions with spans $[10^{k_L}, \; 10^{-1}]$ and $[10^{k_V}, \; 10^{-1}]$, respectively. The cell death rates $(d_S,\, d_E ,\, d_I ,\, d_L)$ are set to $0.04 \ \text{hr}^{-1}$ in e and $0.20 \ \text{hr}^{-1}$ in f. The *x* and *y* axis mark $10^{k_L}$ and $10^{k_V}$, respectively. The dotted and dashed lines represent the lower thresholds for an obligate lytic virus ($p = 0, \gamma = 0\ \text{hr}^{-1}$) to survive 100 growth cycles with probabilities of 0.05 and 0.95, respectively. All other simulation parameters can be found in Table S1 and Table S2.

Thus far, we have established how temperate strategies evolve and allow for viral persistence in response to short-term and long-term selection pressures in deterministically periodic environments. However, viruses also encounter environmental stochasticity—in the present context this could be represented by changing the duration of growth cycles, the filtration conditions, or both. To explore the impact of environmental stochasticity on long-term viral strategies, we simulate 100 growth cycles of 24 hr each with 10 per cent lysogens passaging from one cycle to the next while randomly selecting a fraction *q_V_* of virions to be passaged from a log uniform distribution with a span $[10^{-10},10^{-1}]$ ( Figure 6a). We find that both obligately lytic and obligately lysogenic strategies lead to viral extinction after a few cycles (Figure 6b, d). The obligately lysogenic virus dies out because it does not produce enough viral copies to sustain the virus population across cycles. The obligately lytic virus, on the other hand, dies out more slowly. Local extinction of the obligately lytic virus occurs when stochastic fluctuations generate a series of filtration steps with very low values of $q_V$ (Figure 6b). The temperate virus, however, overcomes both these problems—through horizontal transmission it produces enough viral copies to prevent gradual extinction and through vertical transmission, it maintains enough lysogens that serial passage conditions representing high virion decay (i.e. low $q_V$ values) do not impact the viral population significantly (Figure 6c). We find similar scenarios for the high cell death rate case when $q_V$ is drawn a log-uniform distribution with the span $[10^{-5}, 10^{-1}]$ (Figure S13).

We extend this analysis further to investigate how the long-term survival probability of a virus changes with respect to the span of fluctuations in $q_L$ and $q_V$. For this analysis, we simulate 100 different realizations tracking 100 growth cycles for a one host–one virus system, where the virus is either obligately lytic, temperate, or obligately lysogenic (see section 2.3 for simulation details). Figure 6e shows the survival probability for a temperate virus that maximized the steady-state viral genome densities given lysogen-only filtration conditions (Figure 4c). The temperate strategy persists in extreme environments for either lysogens or virions, but is unable to persist in circumstances that are adverse for both lysogens and virions. While the temperate virus does not survive in some environments where obligately lytic viruses survive, it can also survive in environments that may be hostile to virions but good for cells (Figure 6e). In the $[k_L,\ k_V]$ domain presented in Figure 6e, the obligately lysogenic virus strategy never survives, since its survival is strongly dependent on a large fraction of the lysogens being in the filtrate. Again, in Figure 6f we obtain similar results regarding survival for the high cell death rate case (temperate phage strategy features shown in Figure S10). We do note that exogenous death rates matter, e.g. in this case there is a narrower range of fluctuations in which obligately lytic and temperate strategies can survive (Figure 6e). These findings further reinforce the evolutionary benefits of being temperate in both deterministically (Figure 3) and stochastically (Figure 6) fluctuating environments.

## 4. Discussion

In this study, we showed how long-term reproduction pressures (set by periodic differential dilution during filtration) acting in conflict with short-term reproduction pressures (set by the host and nutrient availability) favour the emergence of temperate strategies that include both lysis and lysogeny. In particular, we showed that even if an obligately lytic virus produces more offspring over a short timescale, a temperate virus might do better on a longer timescale due to the higher survival rate of lysogens. We showed that across a continuum of traits between lysis and latency, strategies that balance the exploitation of hosts in the short term and maintenance of lysogen populations in the long term persist while obligately lytic and obligately lysogenic strategies go locally extinct. Changing the environmental context changes the eco-evolutionary outcomes of the temperate phage-host system. More latent strategies are favoured when the growth cycle periods are shorter and when lysogens are passaged across growth cycles. However, lytic strategies are favoured when the growth cycle periods are longer and when virions are passaged across growth cycles. Finally, temperate strategies provide resilience against environmental stochasticity. In scenarios with fixed lysogen mortality but variable virion mortality, obligately lysogenic and obligately lytic strategies are more susceptible to population crashes than temperate strategies.

The present findings extend previous work which used a cell-centric measure of fitness to identify conditions that favoured latency, both in host-only conditions and in scenarios with an endemic lytic phage (Weitz et al., 2019; Li et al., 2020; Berngruber et al., 2013; Gandon, 2016). These studies showed that low host availability and high phage mortality favour latency. However, the cell-centric reproduction number provides information about the short-term success of a viral strategy at the scale of an individual infected cell. In contrast, (Wahl et al., 2019) used analysis of steady states to identify conditions that allowed for long-term persistence of temperate phage, showing that strong negative feedback between phage and host densities favoured latency while mechanisms that decoupled host density from phage density favoured lysis. Our framework allows for evaluating the realistic scenario (extending the work of (Maslov and Sneppen, 2015)) that external drivers (periodic host input and differential dilution) can lead to conflicting selection pressures across timescales.

These findings came with several important caveats. For example, the present model does not incorporate phenotypic plasticity in the lysis-lysogeny switch. Across phage-bacteria systems, the probability of lysogen formation as well as the rate of prophage induction can depend on the physiological state of the host. As nutrients become scarce, host metabolism slows down, which in turn slows the infection cycle; the latent period of infection increases and burst size decreases as nutrient availability reduces (Hadas et al., 1997; Abedon et al., 2001; Wang, 2006; Bruce et al., 2021; Gandon, 2016). These changes might favour lysogeny. On the other hand, adsorption and viral lysis rates are known to increase with increased resource availability (Choua and Bonachela, 2019; Bonachela, 2024). As a result, feedback mechanisms that enhance resource availability, such as the viral shunt, would favour lysis. The lysis-lysogeny switch is also sensitive to virus-host ratios, which can be sensed either through secreted molecules such as arbitrium (Erez et al., 2017; Shivam et al., 2022) or through cellular multiplicity of infection (Kourilsky, 1973; Golding, 2016; Zhang et al., 2021). Since the nutrient, host, and viral densities are tightly coupled during growth cycles, phenotypic plasticity may play an important role in modulating population dynamics and evolution of viral traits (Gandon, 2016; Choua and Bonachela, 2019; Lion and Gandon, 2022; Bruce et al., 2021). This plasticity might also explain why the viral genome density maximizing strategies we obtained in Figure 4e have integration probabilities higher than those typically reported in experiments. Zeng et al. (Zeng et al., 2010) report integration probabilities between 0.2 and 0.4 for singly infected cells and between 0.6 and 0.8 for cells with 5 coinfections, which are consistent with the magnitude of the evolutionarily stable integration probabilities presented in Figure 5e. We note that the steady viral genome density maximizing induction rates ($\gamma^*$ values) reported in Figure 4f are significantly larger than those reported for phage *λ* (Little and Michalowski, 2010). We note that the current model does not include plastic responses, e.g. in which induction rates increase above 0.1/hr in stressful conditions. Instead, we note that model-inferred induction rates can be as low as 10^−3^/hr, consistent with measurements of *in vivo* induction rates (De Paepe et al., 2016). Nevertheless, our results suggest that temperate strategies are beneficial, even in the absence of any plasticity in response to internal/external cues regarding host or resource availability. Extending the present eco-evolutionary model to incorporate phenotypic plasticity and/or signalling cues would allow a detailed exploration of conflicts of selection across time scales, as well as a means to evaluate the evolutionary benefits of plastic temperate strategies. In doing so, the use of a serial passage setup could make such studies experimentally tractable.

In closing, leveraging a serial passage model of the eco-evolutionary dynamics of temperate phage, we have shown how short-term selection pressures to reproduce during boom periods and long-term selection pressures to persist in bust periods can work in opposition, leading to the emergence of evolutionarily stable temperate strategies involving a mix of both lysis and lysogeny. In doing so, we also identified how ecological drivers, including the duration of growth cycles and the severity of bust periods, can tilt the balance towards strategies with more lysogenic-like traits or more lytic-like traits. The emergence of intermediate temperate phenotypes and the complexity of feedback during growth periods also point to directions that are likely amenable to further theoretical and experimental study: exploring how plasticity in viral strategies modulates ecological dynamics in the short-term and how this plasticity evolves in the long-term.

## Supplementary Material

veaf019_Supp

## Data Availability

There is no experimental data associated with this manuscript. All code associated with the manuscript was written in MATLAB 2022b and MATLAB 2023b and is available at https://github.com/tapangoel1994/EcoEvoDynamicsInPeriodicEnvironments and is archived on Zenodo (Goel et al., 2025).
