# Supplementary material for: Eco-evolutionary dynamics of temperate phages in periodic environments
Source: Virus Evol. 2025 Apr 29;11(1):veaf019. doi: 10.1093/ve/veaf019 (PMC12105577; doi:10.1093/ve/veaf019)
Supplement: veaf019_Supp [file veaf019_supp.zip › veaf019_Supp_AllFiguresTables.pdf]

Eco-evolutionary dynamics of temperate phages in periodic environments

Tapan Goel[1,*,✉], Stephen J. Beckett[1,2,*], Joshua S. Weitz[1,2,3,†,*,✉]

1 Department of Biology, University of Maryland, College Park, MD, USA
2 University of Maryland Institute for Health Computing, North Bethesda, MD, USA
3 Department of Physics, University of Maryland, College Park, MD, USA
* Previous address: School of Biological Sciences, Georgia Institute of Technology, Atlanta, GA, USA
† Previous address: Institut de Biologie, École Normale Supérieure, Paris, France
✉email: tgoel36@umd.edu, jsweitz@umd.edu

Supplementary Material

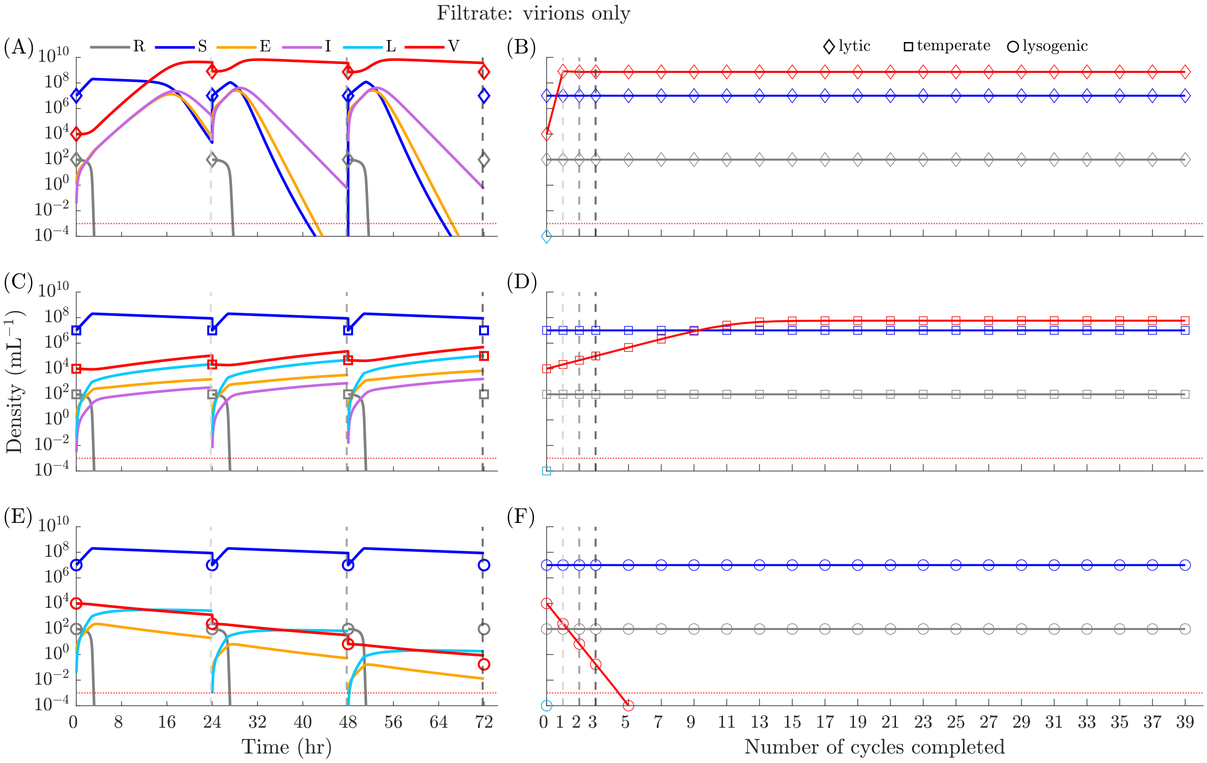

Figure S1. Population dynamics when only virions pass through the filter: Population dynamics over the first three 24-hr growth cycles for (A) obligately lytic ($p = 0, \gamma = 0$ hr^{-1}) ($\diamond$), (C) temperate ($p = 0.92, \gamma = 0.006$ hr^{-1}) ($\square$) and (E) obligately lysogenic ($p = 1, \gamma = 0$ hr^{-1}) ($\circ$) viruses when only virions pass through the filter ($q_V = 0.2$). Population densities at the beginning of each growth cycle for (B) obligately lytic ($p = 0, \gamma = 0$ hr^{-1}) ($\diamond$), (D) temperate ($p = 0.92, \gamma = 0.006$ hr^{-1}) ($\square$) and (F) lysogenic ($p = 1, \gamma = 0$ hr^{-1}) ($\circ$) viruses when only virions pass through the filter. The dashed vertical lines at the 24 hr, 48 hr and 72 hr marks in plots A, C and E correspond to the dashed vertical lines at cycle number 1, 2 and 3 in plots B, D and F respectively. Dotted horizontal lines in A-F represent the critical density threshold $\epsilon = 10^{-3}$ mL^{-1}. All other simulation parameters can be found in Table S1 and Table S2.

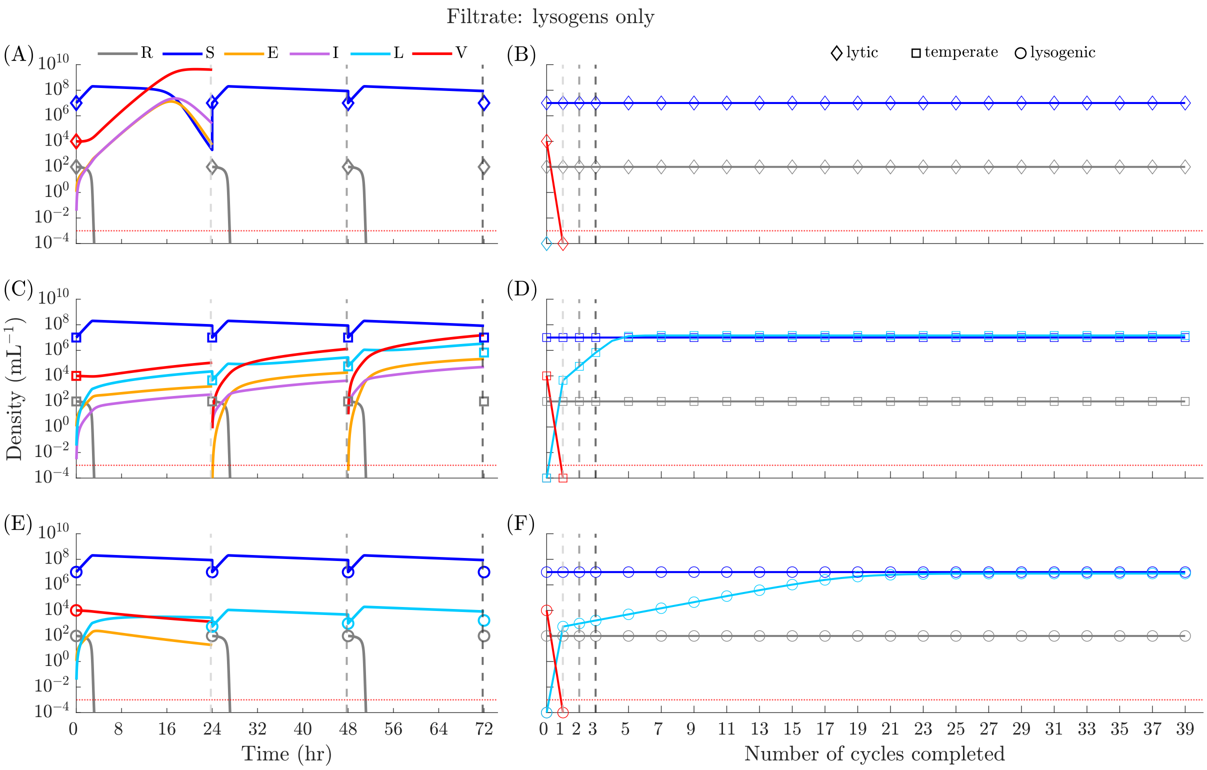

Figure S2. Population dynamics when only lysogens pass through the filter: Population dynamics over the first three 24-hr growth cycles for (A) obligately lytic ($p = 0, \gamma = 0$ hr^{-1}) ($\diamond$), (C) temperate ($p = 0.92, \gamma = 0.006$ hr^{-1}) ($\square$) and (E) obligately lysogenic ($\gamma = 0$ hr$^{-1}, p = 1$) ($\circ$) viruses when only lysogens pass through the filter ($q_L = 0.2$). Population densities at the beginning of each growth cycle for (B) obligately lytic ($p = 0, \gamma = 0$ hr^{-1}) ($\diamond$), (D) temperate ($p = 0.92, \gamma = 0.006$ hr^{-1}) ($\square$) and (F) lysogenic ($p = 1, \gamma = 0$ hr^{-1}) ($\circ$) viruses when only lysogens pass through the filter. The dashed vertical lines at the 24 hr, 48 hr and 72 hr marks in plots A, C and E correspond to the dashed vertical lines at cycle number 1, 2 and 3 in plots B, D and F respectively. Dotted horizontal lines in A-F represent the critical density threshold $\epsilon = 10^{-3}$ mL^{-1}. All other simulation parameters can be found in Table S1 and Table S2.

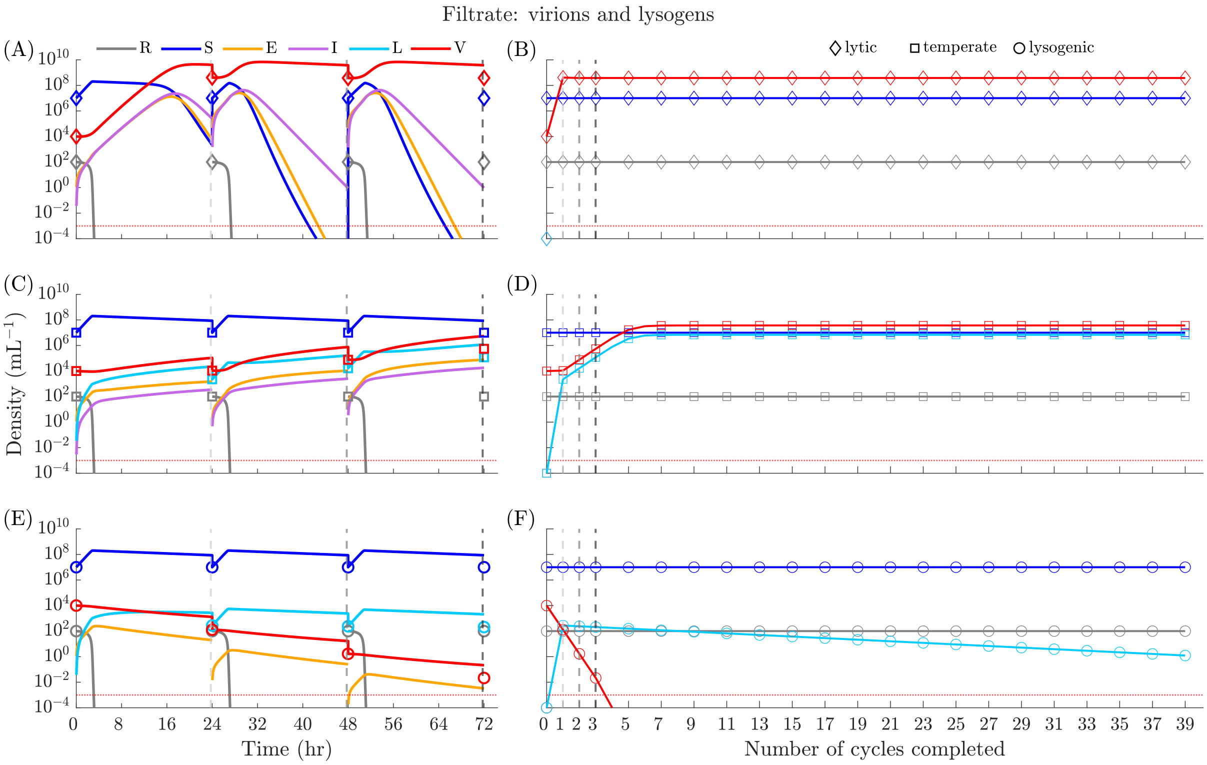

Figure S3. Population dynamics when virions and lysogens pass through the filter: Population dynamics over the first three 24-hr growth cycles for (A) obligately lytic ($p = 0, \gamma = 0$ hr^{-1}) (◇), (C) temperate ($p = 0.92, \gamma = 0.006$ hr^{-1}) (□) and (E) obligately lysogenic ($p = 1, \gamma = 0$ hr^{-1}) (○) viruses when virions and lysogens pass through the filter ($q_L = 0.1, q_V = 0.1$). Population densities at the beginning of each growth cycle for (B) obligately lytic ($p = 0, \gamma = 0$ hr^{-1}) (◇), (D) temperate ($p = 0.92, \gamma = 0.006$ hr^{-1}) (□) and (F) lysogenic ($p = 1, \gamma = 0$ hr^{-1}) (○) viruses when virions and lysogens pass through the filter. The dashed vertical lines at the 24 hr, 48 hr and 72 hr marks in plots A, C and E correspond to the dashed vertical lines at cycle number 1,2 and 3 in plots B, D and F respectively. Dotted horizontal lines in A-F represent the critical density threshold $\epsilon = 10^{-3}$ mL^{-1}. All other simulation parameters can be found in Table S1 and Table S2.

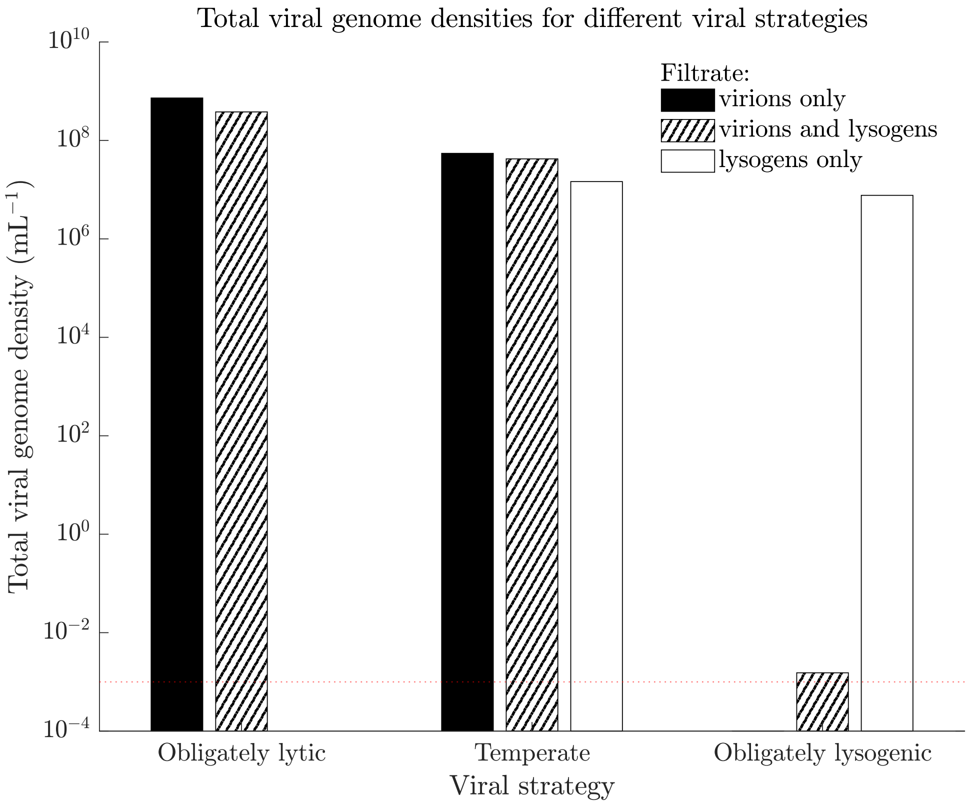

Figure S4. Steady state viral genome densities across strategies and filtration conditions: Bar chart showing total viral genome densities at steady state for obligately lytic ($p = 0, \gamma = 0$ hr^{-1}), temperate ($p = 0.92, \gamma = 0.006$ hr^{-1}) and obligately lysogenic ($p = 1, \gamma = 0$ hr^{-1}) viruses subject to 24-hr growth cycles under different filtration conditions: only virions in the filtrate ($q_V = 0.2$, solid), virions and lysogens in the filtrate ($q_L = 0.1, q_V = 0.1$, striped) and, only lysogens in the filtrate ($q_L = 0.2$, unfilled). All other simulation parameters are given in Table S1 and Table S2. Dotted horizontal line represents the critical density threshold $\epsilon = 10^{-3}$ mL^{-1}.

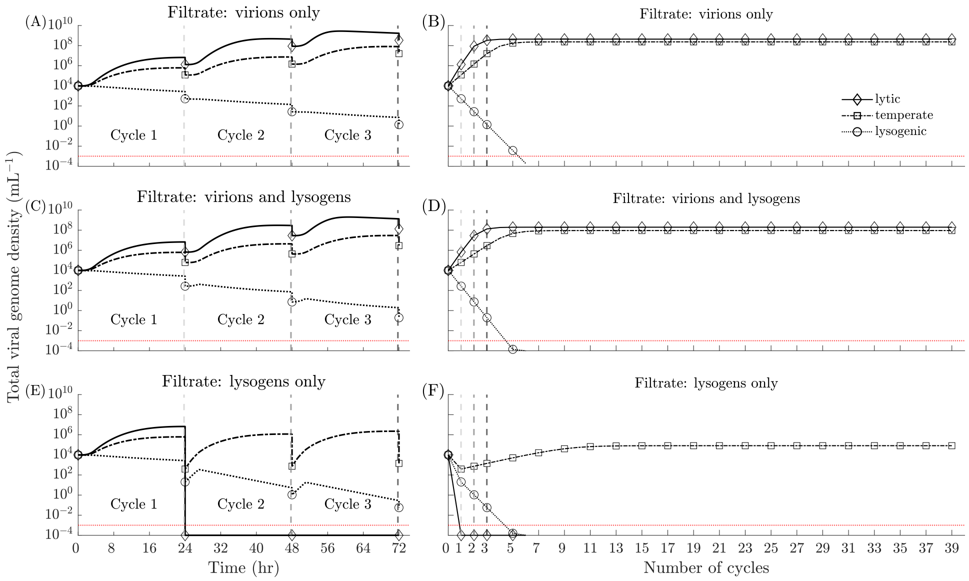

Figure S5. Long-term population dynamics: Total viral genome densities over the first three 24-hr growth cycles for obligately lytic ($p = 0, \gamma = 0$ hr^{-1}) ($\diamond$), temperate ($p = 0.5, \gamma = 0.083$ hr^{-1}) ($\square$) and lysogenic ($p = 1, \gamma = 0$ hr^{-1}) ($\circ$) viruses when (A) only virions ($q_V = 0.2$), (C) lysogens and virions ($q_V = 0.1, q_L = 0.1$) and, (E) only lysogens ($q_L = 0.2$) pass through the filter. Total viral genome densities at the beginning of each growth cycle for obligately lytic ($p = 0, \gamma = 0$ hr^{-1}) ($\diamond$), temperate ($p = 0.5, \gamma = 0.083$ hr^{-1}) ($\square$) and lysogenic ($p = 1, \gamma = 0$ hr^{-1}) ($\circ$) viruses when (B) only virions, (D) lysogens and virions and, (F) only lysogens pass through the filter. The dashed vertical lines at the 24 hr, 48 hr and 72 hr marks in plots A, C and E correspond to the dashed vertical lines at cycle number 1, 2 and 3 in plots B, D and F respectively. Cell death rates (d_S, d_E, d_I, d_L) are set to 0.20 hr^{-1}. All other simulation parameters are given in Table S1 and Table S2. Dotted horizontal lines in A-F represent the critical density threshold $\epsilon = 10^{-3}$ mL^{-1}.

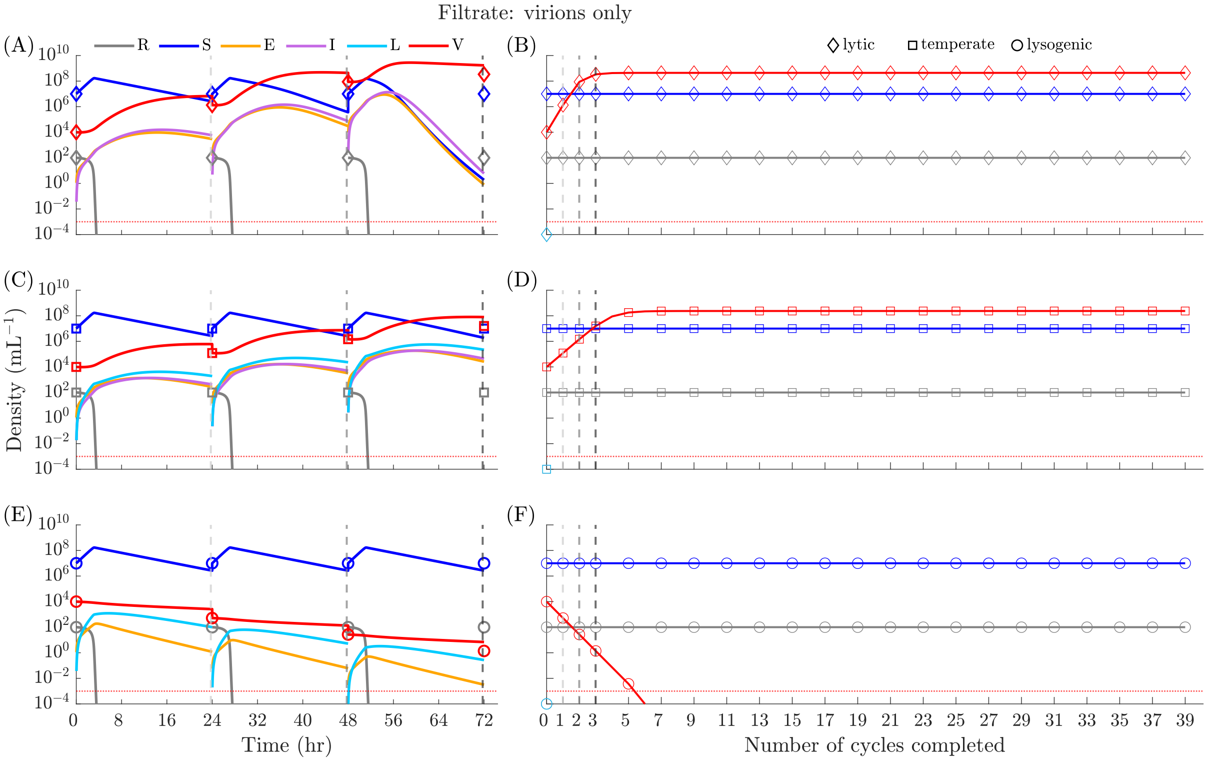

Figure S6. Population dynamics when only virions pass through the filter: Population dynamics over the first three 24-hr growth cycles for (A) obligately lytic ($p = 0, \gamma = 0$ hr^{-1}) ($\diamond$), (C) temperate ($p = 0.5, \gamma = 0.083$ hr^{-1}) ($\square$) and (E) obligately lysogenic ($p = 1, \gamma = 0$ hr^{-1}) ($\circ$) viruses when only virions pass through the filter ($q_V = 0.2$). Population densities at the beginning of each growth cycle for (B) obligately lytic ($p = 0, \gamma = 0$ hr^{-1}) ($\diamond$), (D) temperate ($p = 0.5, \gamma = 0.083$ hr^{-1}) ($\square$) and (F) lysogenic ($p = 1, \gamma = 0$ hr^{-1}) ($\circ$) viruses when only virions pass through the filter. The dashed vertical lines at the 24 hr, 48 hr and 72 hr marks in plots A, C and E correspond to the dashed vertical lines at cycle number 1, 2 and 3 in plots B, D and F respectively. Cell death rates (d_S, d_E, d_I, d_L) are set to 0.20 hr^{-1}. All other simulation parameters are given in Table S1 and Table S2. Dotted horizontal lines in A-F represent the critical density threshold $\epsilon = 10^{-3}$ mL^{-1}.

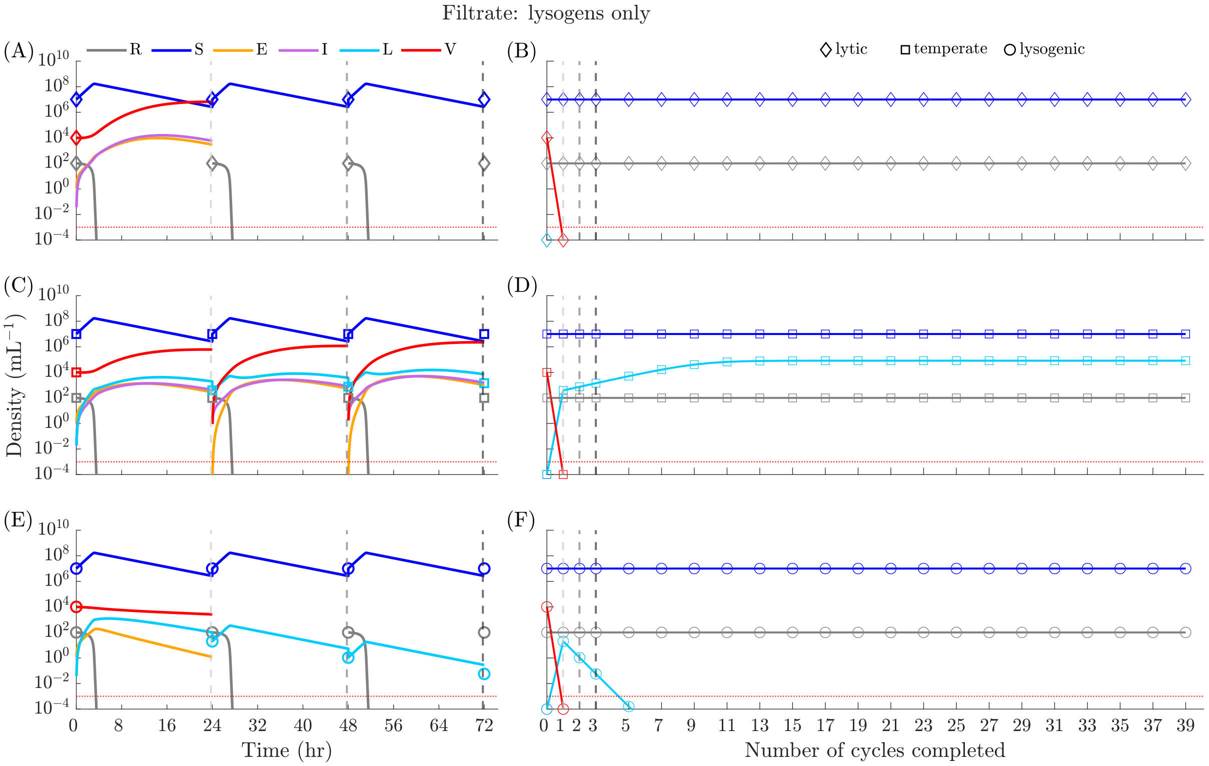

Figure S7. Population dynamics when only lysogens pass through the filter: Population dynamics over the first three 24-hr growth cycles for (A) obligately lytic ($p = 0, \gamma = 0$ hr^{-1}) ($\diamond$), (C) temperate ($p = 0.5, \gamma = 0.083$ hr^{-1}) ($\square$) and (E) obligately lysogenic ($\gamma = 0$ hr$^{-1}, p = 1$) ($\circ$) viruses when only lysogens pass through the filter ($q_L = 0.2$). Population densities at the beginning of each growth cycle for (B) obligately lytic ($p = 0, \gamma = 0$ hr^{-1}) ($\diamond$), (D) temperate ($p = 0.5, \gamma = 0.083$ hr^{-1}) ($\square$) and (F) lysogenic ($p = 1, \gamma = 0$ hr^{-1}) ($\circ$) viruses when only lysogens pass through the filter. The dashed vertical lines at the 24 hr, 48 hr and 72 hr marks in plots A, C and E correspond to the dashed vertical lines at cycle number 1, 2 and 3 in plots B, D and F respectively. Cell death rates (d_S, d_E, d_I, d_L) are set to 0.20 hr^{-1}. All other simulation parameters are given in Table S1 and Table S2. Dotted horizontal lines in A-F represent the critical density threshold $\epsilon = 10^{-3}$ mL^{-1}.

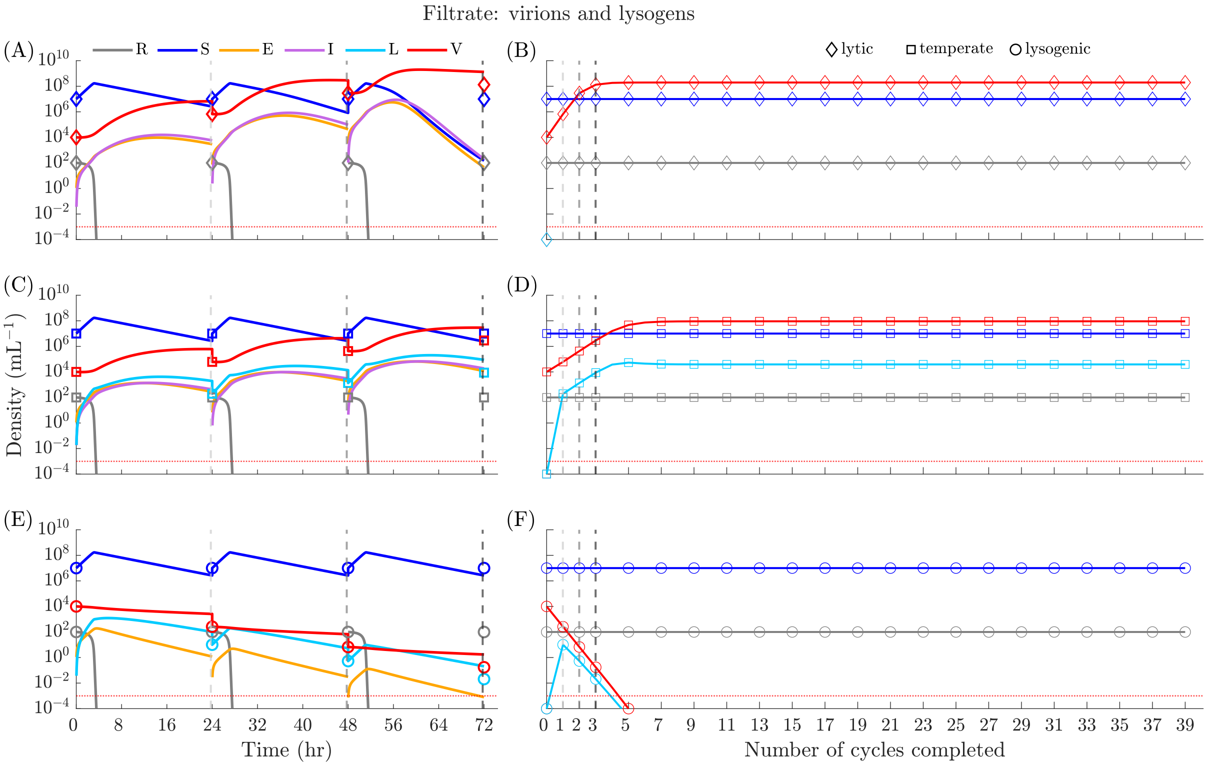

Figure S8. Population dynamics when virions and lysogens pass through the filter: Population dynamics over the first three 24-hr growth cycles for (A) obligately lytic ($p = 0, \gamma = 0$ hr^{-1}) ($\diamond$), (C) temperate ($p = 0.5, \gamma = 0.083$ hr^{-1}) ($\Box$) and (E) obligately lysogenic ($p = 1, \gamma = 0$ hr^{-1}) ($\circ$) viruses when virions and lysogens pass through the filter ($q_L = 0.1, q_V = 0.1$). Population densities at the beginning of each growth cycle for (B) obligately lytic ($p = 0, \gamma = 0$ hr^{-1}) ($\diamond$), (D) temperate ($p = 0.5, \gamma = 0.083$ hr^{-1}) ($\Box$) and (F) lysogenic ($p = 1, \gamma = 0$ hr^{-1}) ($\circ$) viruses when virions and lysogens pass through the filter. The dashed vertical lines at the 24 hr, 48 hr and 72 hr marks in plots A, C and E correspond to the dashed vertical lines at cycle number 1, 2 and 3 in plots B, D and F respectively. Cell death rates (d_S, d_E, d_I, d_L) are set to 0.20 hr^{-1}. All other simulation parameters are given in Table S1 and Table S2. Dotted horizontal lines in A-F represent the critical density threshold $\epsilon = 10^{-3}$ mL^{-1}.

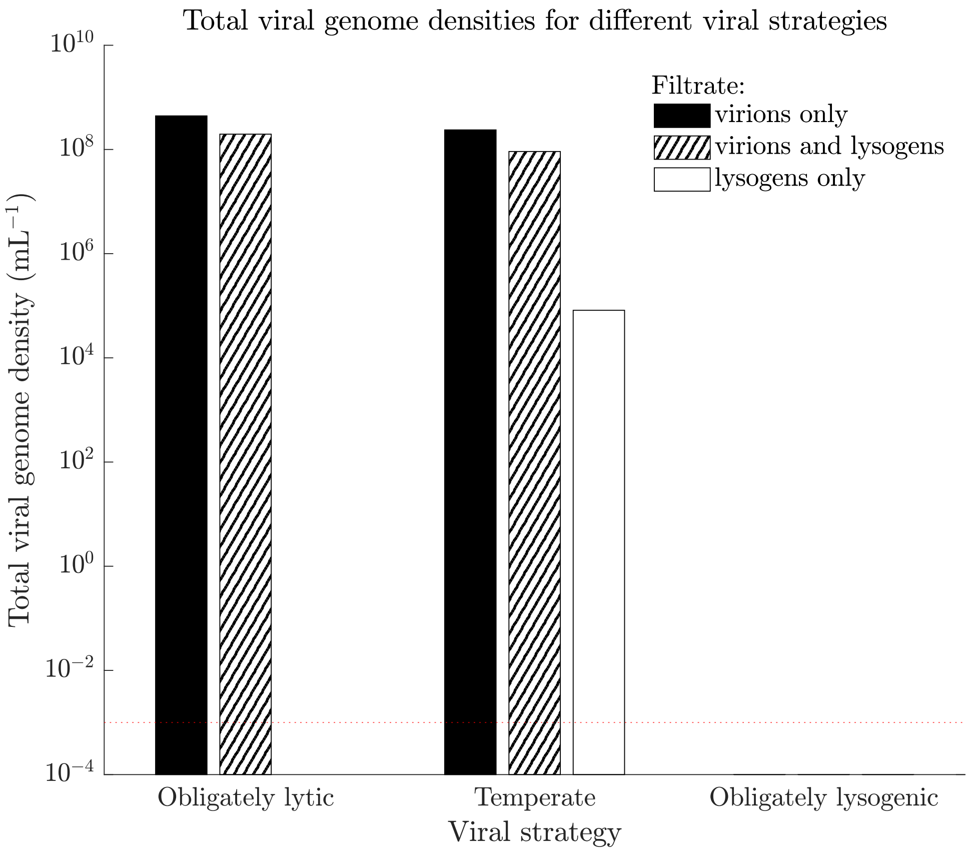

Figure S9. Steady state viral genome densities across strategies and filtration conditions: Bar chart showing total viral genome densities at steady state for obligately lytic ($p = 0, \gamma = 0$ hr^{-1}), temperate ($p = 0.5, \gamma = 0.083$ hr^{-1}) and obligately lysogenic ($p = 1, \gamma = 0$ hr^{-1}) viruses subject to 24-hr growth cycles under different filtration conditions: only virions in the filtrate ($q_V = 0.2$, solid), virions and lysogens in the filtrate ($q_L = 0.1, q_V = 0.1$, striped) and, only lysogens in the filtrate ($q_L = 0.2$, unfilled). Cell death rates (d_S, d_E, d_I, d_L) are set to 0.20 hr^{-1}. All other simulation parameters are given in Table S1 and Table S2. Dotted horizontal line represents the lower critical density threshold $\epsilon = 10^{-3}$ mL^{-1}.

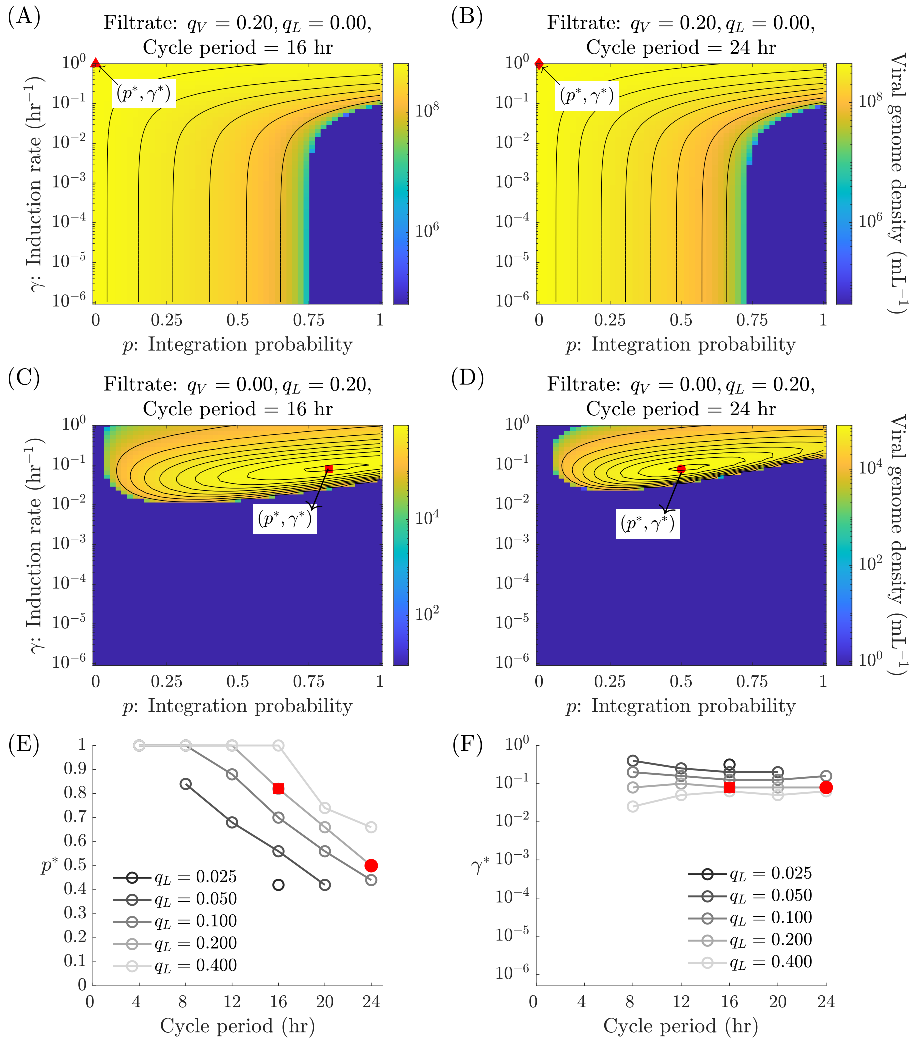

Figure S10. Steady states for different long-term selection pressures: Heatmaps of the density of viral genome copies at the beginning of a steady state growth cycle across different induction rates and integration probabilities when only virions ($q_V = 0.2$) are passaged every (A) 16 hr and every (B) 24 hr, and when only lysogens ($q_L = 0.2$) are passaged every (C) 16 hr and every (D) 24 hr. (p^*, γ^*) denote the strategies that maximize the steady state viral genome density for the given filtration and cycle period conditions.(E) Integration probabilities and (F) induction rates of strategies that maximize the steady state total viral genome density as a function of cycle period. Line colors correspond to the fraction of lysogens that pass from one cycle to the next. Fraction of virions in the filtrate (q_V) is set to zero. The red squares in C, E and F correspond to the same strategy for the same filtration and cycle period conditions. Similarly, the red circles in D, E and F correspond to the same strategy. Cell death rates (d_S, d_E, d_I, d_L) are set to 0.20 hr^{-1}. All other simulation parameters are given in Table S1 and Table S2.

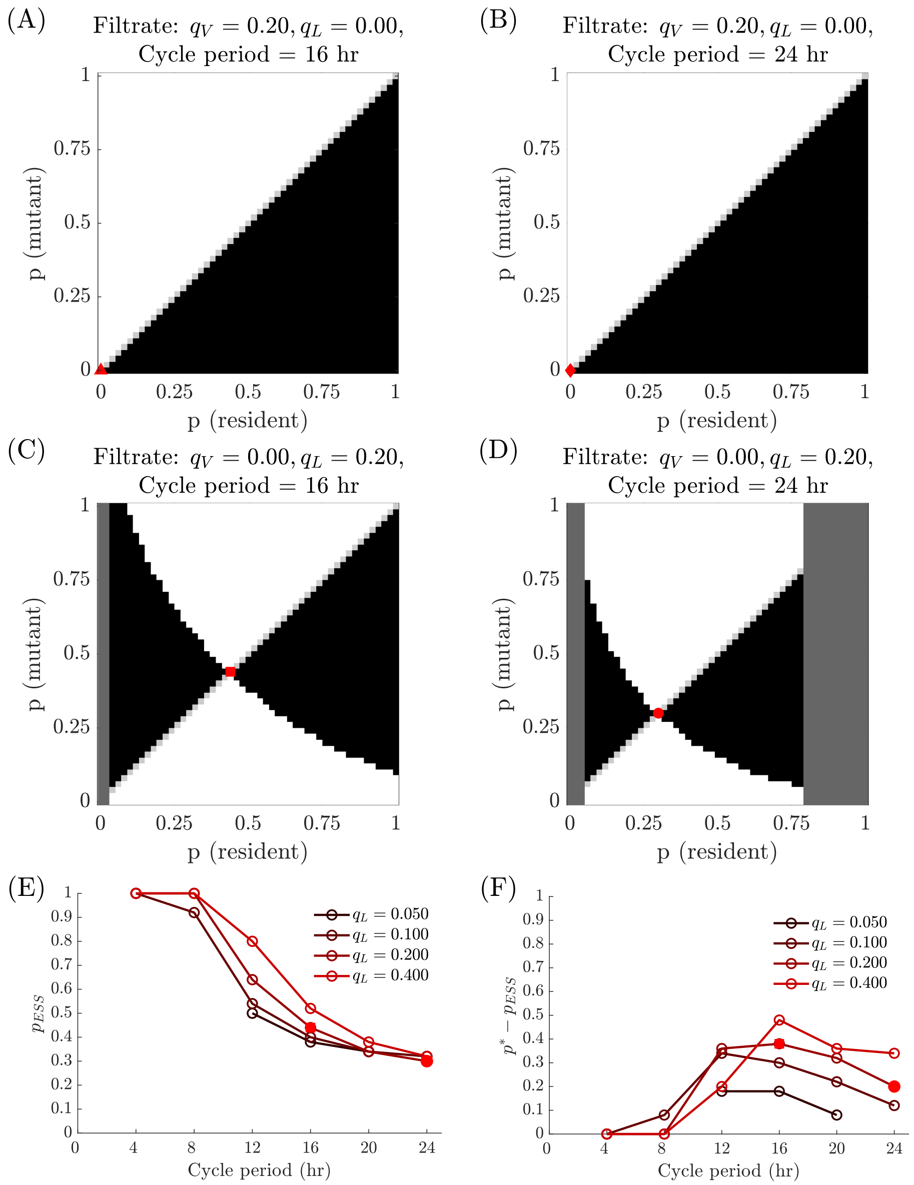

Figure S11. Invasion analysis: Pairwise invasibility plots when only virions ($q_V = 0.2$) are passaged every (A) 16 hr and every (B) 24 hr, and when only lysogens ($q_L = 0.2$) are passaged every (C) 16 hr and every (D) 24 hr. Red markers indicate the evolutionarily stable integration probability (p_{ESS}) for fixed induction rates. Induction rates are fixed to maximize viral genome density at steady state ($\gamma = \gamma^*$) for each condition. Dark gray regions correspond to integration probabilities at which the resident virus does not persist in the one host-one virus system (see section 2.3 in the main text for details). Light gray marks regions where the resident and mutant have identical life history strategies. (E) Evolutionarily stable integration probability as a function of cycle period. Colors correspond to the fraction of lysogens that pass from one cycle to the next. ESS for each condition is calculated by setting the induction rate to be the viral genome density maximizing induction rate for that condition. (F) Difference between the steady state viral genome maximizing integration probability and the evolutionarily stable integration probability for different cycle periods and filtration conditions. The red squares in C, E and F correspond to the same strategy for the same filtration and cycle period conditions. Similarly, the red circles in D, E and F correspond to the same strategy. Cell death rates (d_S, d_E, d_I, d_L) are set to 0.20 hr^{-1}. All other simulation parameters are given in Table S1 and Table S2.

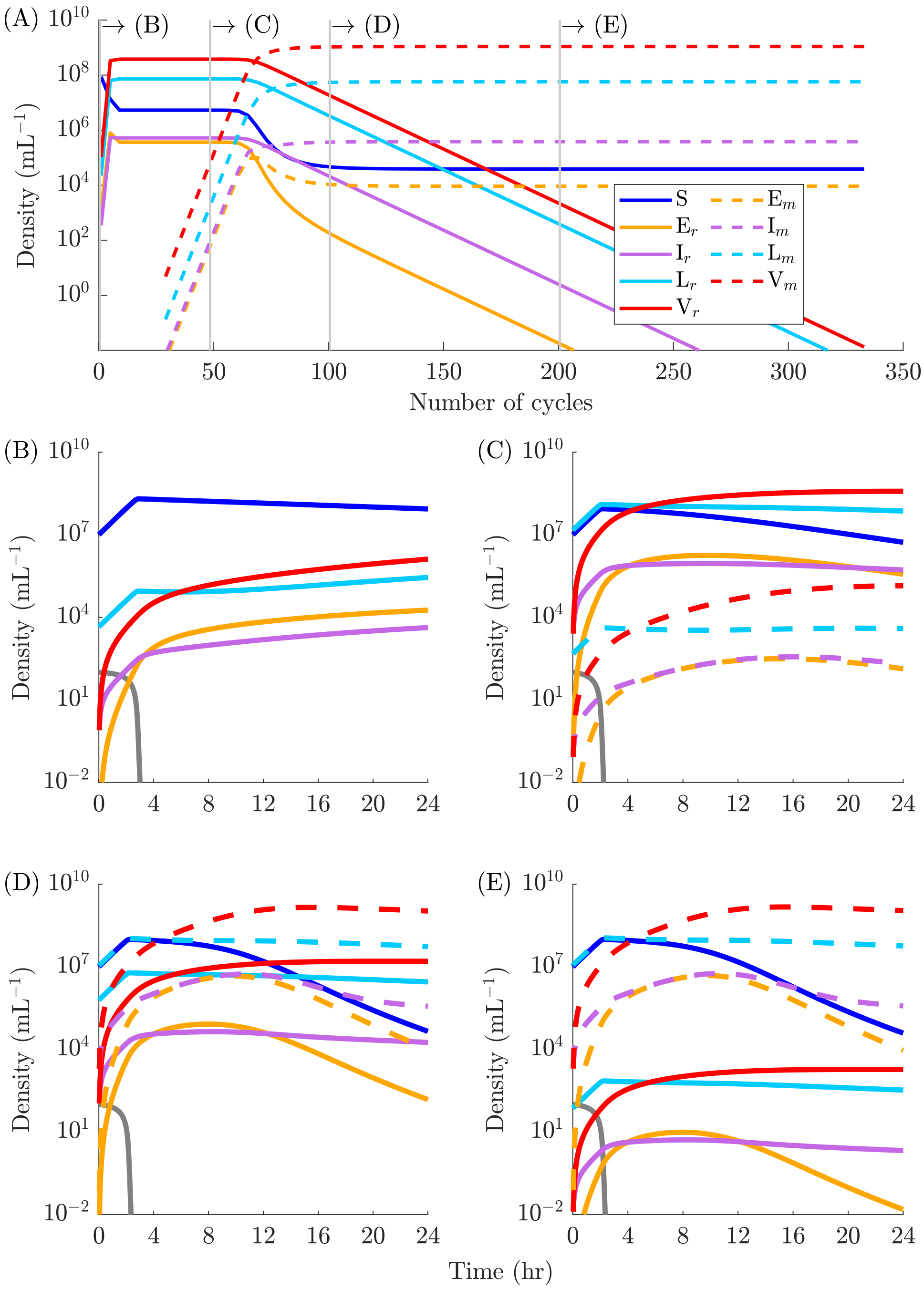

Figure S12. Cycle-to-cycle invasion dynamics: (A) Final cell/virion densities at the end of each cycle for (r)esident ($p^* = 0.92, \gamma^* = 0.0063$ hr^{-1}) and (m)utant ($p_{\text{ESS}} = 0.42, \gamma = 0.0063$ hr^{-1}) virus types. The growth cycle duration is 24 hr, and only 20% of lysogens ($q_L = 0.2$) from one growth cycle pass to the next growth cycle. Gray vertical lines highlight the 1st, 49th, 101st and 201st growth cycles, the within cycle dynamics for which are shown in panels (B), (C), (D) and (E) respectively. All other simulation parameters are given in Table S1 and Table S2.

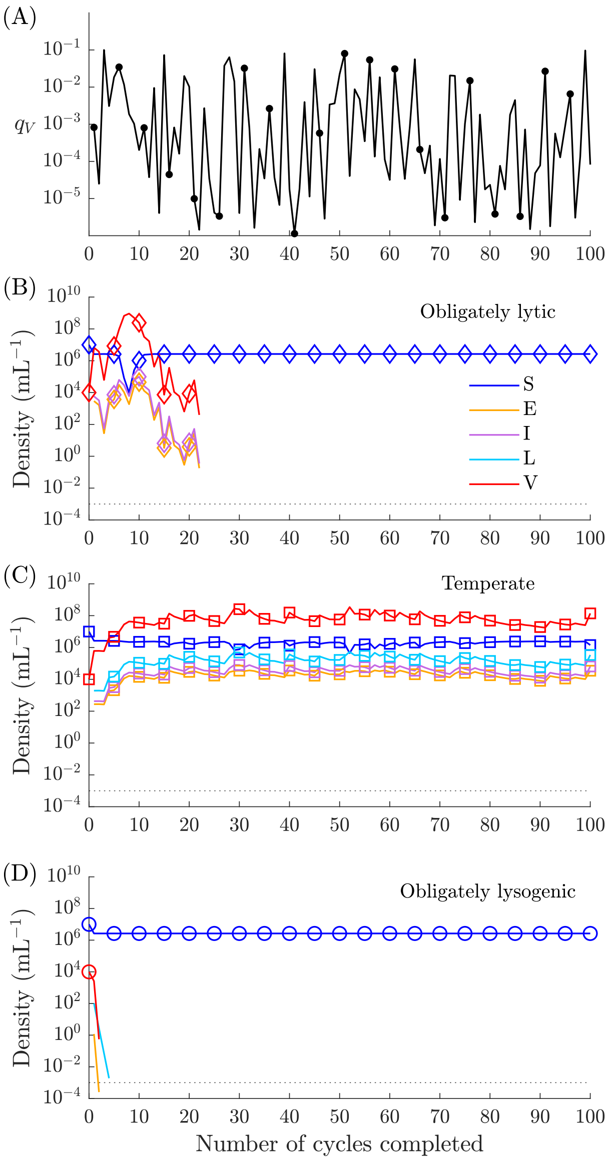

Figure S13. Population dynamics with stochastic filtration: (A) q_V values for 100 growth cycles, chosen from a log uniform distribution over the span $[10^{-5}, 10^{-1}]$. Population densities at the end of each growth cycle for (B) obligately lytic ($p = 0, \gamma = 0$ hr^{-1}), (C) temperate ($p = 0.5, \gamma = 0.083$ hr^{-1}) and (D) obligately lysogenic ($p = 1, \gamma = 0$ hr^{-1}) viruses across hundred 24-hr cycles. 10% lysogens ($q_L = 0.1$) are passaged from one cycle to the next in every case, but the virion fraction passaged from cycle-to-cycle is set by q_V shown in (A). Markers at every 5th cycle denote that the lines represent discrete cycle-to-cycle dynamics and not continuous time dynamics. Cell death rates (d_S, d_E, d_I, d_L) are set to 0.20 hr^{-1}. All other simulation parameters are given in Table S1 and Table S2.

Tables

Parameter name	Parameter symbol	Value	Units
Conversion efficiency	e	5×10^{-7}	μg/cell
Maximal cell growth rate	μ_{max}	1.2	hr^{-1}
Half velocity constant	R_{in}	4.0	μg/mL
Death rate of susceptible host	d_S	0.04	hr^{-1}
Death rate of exposed cells	d_E	0.04	hr^{-1}
Death rate of lysogens	d_L	0.04	hr^{-1}
Death rate of lytically infected cells	d_I	0.04	hr^{-1}
Transition rate	λ	2	hr^{-1}
Lysis rate	η	1	hr^{-1}
Burst size	β	50	
Adsorption rate	ϕ	3.4 $\times 10^{-10}$	mL/hr
Viral decay rate	m	1/24	hr^{-1}
Integration probability	p	$\{0, 0.02, 0.04 \text{ ... } 0.98, 1\}$	
Induction rate	γ	$\{10^{-6}, \text{ ... } 10^{-0.9}, 10^{-1}\}$	hr^{-1}

Table S1. SEILV model parameters (see Table A1 in (Shivam et al., 2022)).

Parameter name	Parameter symbol	Value	Units
Initial resource density	R_0	100	μg/mL
Initial host density	S_0	10^7	cells/mL
Initial virion density	V_0	10^4	virions/mL
Initial virion density (resident)	V_{a0}	10^4	virions/mL
Initial viral genome density (mutant)	M	10ϵ	genomes/mL
Fraction of resources in filtrate	q_R	0	
Fraction of susceptible hosts in filtrate	q_S	0	
Fraction of exposed cells in filtrate	q_E	0	
Fraction of lytically infected cells in filtrate	q_I	0	
Fraction of lysogens in filtrate	q_L	$\{0, 0.025, 0.05 \ ... \ 0.2, 0.4\}$	
Fraction of virions in filtrate	q_V	$\{0, 0.05, 0.1, 0.2\}$	
Duration of growth cycle	T	$\{4, 8, \ ... \ 24, 36, 48, 96\}$	hr
Critical density threshold	ϵ	1×10^{-3}	mL^{-1}

Table S2. Initial conditions and filtration conditions for serial passage simulations.
